# Human RNA cap1 methyltransferase CMTr1 cooperates with RNA helicase DHX15 to modify RNAs with highly structured 5′ termini

**DOI:** 10.1098/rstb.2018.0161

**Published:** 2018-11-05

**Authors:** Diana Toczydlowska-Socha, Magdalena M. Zielinska, Malgorzata Kurkowska, Astha, Catarina F. Almeida, Filip Stefaniak, Elzbieta Purta, Janusz M. Bujnicki

**Affiliations:** 1 Laboratory of Bioinformatics and Protein Engineering, International Institute of Molecular and Cell Biology, ul. ks. Trojdena 4, 02-109 Warsaw, Poland; 2 Institute of Molecular Biology and Biotechnology, Faculty of Biology, Adam Mickiewicz University, ul. Umultowska 89, 61-614 Poznan, Poland

**Keywords:** RNA capping, RNA modification, methyltransferase, helicase, CMTr1

## Abstract

The 5′-cap structure, characteristic for RNA polymerase II-transcribed RNAs, plays important roles in RNA metabolism. In humans, RNA cap formation includes post-transcriptional modification of the first transcribed nucleotide by RNA cap1 methyltransferase (CMTr1). Here, we report that CMTr1 activity is hindered towards RNA substrates with highly structured 5′ termini. We found that CMTr1 binds ATP-dependent RNA DHX15 helicase and that this interaction, mediated by the G-patch domain of CMTr1, has an advantageous effect on CMTr1 activity towards highly structured RNA substrates. The effect of DHX15 helicase activity is consistent with the strength of the secondary structure that has to be removed for CMTr1 to access the 5′-terminal residues in a single-stranded conformation. This is, to our knowledge, the first demonstration of the involvement of DHX15 in post-transcriptional RNA modification, and the first example of a molecular process in which DHX15 directly affects the activity of another enzyme. Our findings suggest a new mechanism underlying the regulatory role of DHX15 in the RNA capping process. RNAs with highly structured 5′ termini constitute a significant fraction of the human transcriptome. Hence, CMTr1–DHX15 cooperation is likely to be important for the metabolism of RNA polymerase II-transcribed RNAs.

This article is part of the theme issue ‘5′ and 3′ modifications controlling RNA degradation’.

## 1. Introduction

Cytoplasmic eukaryotic mRNAs and many non-coding RNAs contain a 7-methylguanosine linked to the first transcribed nucleotide via an inverted 5′ to 5′ triphosphate bridge (m^7^GpppN, where N is any nucleotide) [1,2]. This unique molecular structure called cap0 protects capped RNAs from 5′ to 3′ exonuclease cleavage and is essential for the regulation of gene expression, including splicing, nuclear export of mRNA, and translation initiation [3–5]. In many instances, cap0-capped RNAs are modified further, in particular by additional methylations of m^7^G (leading to m^2,2,7^G cap) or methylation of the first few transcribed nucleoside residues (review: [6]). For instance, in higher eukaryotes and in many viruses that replicate in the cytoplasm, the 5′ cap is 2′-*O*-methylated at the first and often also second ribonucleotide residues, yielding cap1 and cap2 structures, respectively [7,8]. CMTr1 and CMTr2 enzymes, responsible for cap1 and cap2 methylations in humans, have been identified and characterized biochemically [9,10].

In humans, cap1 methylation occurs on all capped and polyadenylated RNA molecules, while only about half of these molecules contain cap2 methylation [8]. The roles of these methylations have not been fully elucidated. Nonetheless, the function of cap1 is understood much better than that of cap2 [11]. Cap1 methylation contributes to the recognition and restriction of foreign RNA, particularly in the context of the cell-intrinsic immune response to viruses [12,13]. Indeed, expression of the CMTr1 methyltransferase is augmented by interferon (IFN), supporting a role for differential methylation of RNA cap structures in immune detection and restriction [14].

**Figure 1.**
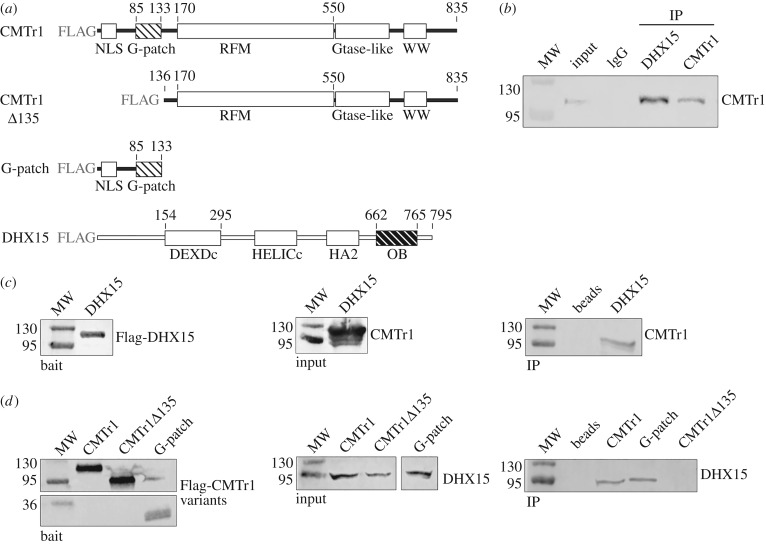
DHX15–CMTr1 interactions. (*a*) Domain structure of human CMTr1 and DHX15 proteins, showing boundaries of structural domains in full-length proteins and in CMTr1 variants used in the assay shown in (*b–d*). Patterned boxes indicate domains responsible for mutual interactions. Co-immunoprecipitation of CMTr1 with endogenous (*b*) and transfected (*c*) DHX15. (*b*) HEK293 lysates were precipitated with anti-DHX15 antibody; the complexes were captured on protein A-conjugated beads and blotted with anti-CMTr1 antibody. (*c*) FLAG-DHX15 vector was transfected into HEK293 cells. Cell lysates were precipitated with anti-FLAG antibody-conjugated agarose and blotted with anti-CMTr1 antibody. (*d*) FLAG-CMTr1 vectors were transfected into HEK293 cells. Cell lysates were precipitated with anti-FLAG antibody-conjugated agarose and blotted with anti-DHX15 antibody. (*c,d*) The left panels show western blotting controls from whole cell lysates from transfected cells to show the levels of expression of the transfected bait proteins. The middle and right panels show western blots with the indicated antibodies before (input) and after immunoprecipitation (IP) using an anti-FLAG antibody, respectively. IgGs (*b*) and empty anti-FLAG agarose beads (*c,d*) were used as negative controls. MW, molecular weight markers.

The structure of the CMTr1 catalytic domain has been determined and shown to constitute a minimal enzymatically active functional element [15]. CMTr1 includes several additional evolutionarily conserved domains, including an N-terminal G-patch domain, which is a short motif with a consensus G-rich sequence implicated in both RNA and protein binding [16] (figure 1*a*). In particular, G-patch domains are known to interact with RNA helicases (review: [17]). While the G-patch domain is not essential for the CMTr1 activity *in vitro*, we hypothesized that it may be responsible for regulating its biological activity in cells.

One of the examples of RNA helicases regulated by G-patch proteins is DHX15 in humans (and its orthologue Prp43 in yeast). It displays only weak helicase activity *in vitro* on DNA/RNA substrates with a single-stranded RNA tail (5′, 3′ or both), and several G-patch-containing proteins can stimulate this activity [18]. For Prp43, as well as DHX15, it was determined that the C-terminal OB-fold domain constitutes the binding site for the G-patch domain [19,20]. DHX15/Prp43 has been implicated thus far in two distinct pathways, RNA splicing and ribosome biogenesis [21]. A number of studies reported regulation of the ATPase and/or helicase activity of DHX15/Prp43 by various G-patch proteins. Known regulators of DHX15 in RNA splicing include TFP11 [22] and RBM5 [23], while regulators of DHX15 in ribosome biogenesis include PINX1 [24] and the NF-κB-repressing factor (NKRF) [20].

Here, we report that CMTr1 acts poorly on capped RNA molecules whose 5′-terminal residues are base-paired to form secondary structure. However, CMTr1 uses its G-patch domain to form a strong complex with DHX15, which significantly improves the efficiency of cap1 methylation of such RNAs. This is the first demonstration of the involvement of DHX15 in post-transcriptional RNA modification, and the first example of a molecular process in which DHX15 directly enables the efficient activity of another enzyme on some of its potential substrates.

## 2. Material and methods

### (a) Cloning

The full-length cDNAs of CMTr1 and DHX15 were obtained from Source BioScience and subcloned into p3xFLAG-CMV^®^-10 vector (Sigma), introducing an N-terminal FLAG-tag, optionally cleavable with PreScission protease, for overexpression in HEK293 cells [10]. For overexpression in bacteria, DHX15 was cloned into the pET28 vector, introducing a C-terminal His-tag. Variants of CMTr1 were constructed using polymerase chain reaction (PCR) with p3xFLAG-CMV10_CMTr1 as a template. DNA construct for expression of the deletion variant that contained the N-terminal part of CMTr1, with the NLS and G-patch domain, was prepared by inserting a stop codon after the triplet coding for Arg133 (forward primer (fv) 5′-TGACAGGAGCTGAACGTGGACTG-3′, reverse primer (rv) 5′-TCACCGGAGTGTCAGACCCAAG-3′). The variant without the G-patch domain was prepared by removing a region that encodes residues 1–135 (fv 5′-GACCAGGAGCTGAACGTGG-3′, rv 5′-GGCGGCCGCAAGCTTGTC-3′).

### (b) Cell culture and transient transfection

FreeStyle 293-F cells (Thermo Fisher Scientific) were grown in suspension in FreeStyle™ 293 Expression Medium (Thermo-Fisher Scientific) in 8% CO_2_ at 37°C. Transient transfections were performed with FectoPro^®^ (Polyplus-transfection) reagent using 0.5 µg of plasmid DNA per 1 ml of suspension culture. Cells were harvested 48 h post-transfection. High Five cells were infected with recombinant baculovirus expressing FLAG-tagged CMTr1 and harvested after 72 h.

### (c) Immunoprecipitation

Cell extracts were prepared in lysis buffer, containing 50 mM Tris–HCl (pH 7.4), 150 mM NaCl, 1 mM EDTA, 0.5% Triton X-100 and a protease inhibitor cocktail for use with mammalian cells and tissue extracts (Sigma). To exclude the effect of RNA-mediated interactions, 10 µg ml^−1^ of RNase A (Sigma) was included in all IP systems. FLAG-IP was performed using anti-FLAG^®^ M2 Affinity Gel (Sigma-Aldrich). Endogenous DHX15 and CMTr1 proteins were precipitated from human embryonic kidney 293 (HEK293) cells homologously expressing recombinant FLAG-tagged variants of CMTr1 and DHX15 proteins, respectively. Lysates were clarified by centrifugation for 30 min at 20 000***g*** and the resulting supernatant was incubated with anti-FLAG antibody-coated beads, with an overnight rotation at 4°C. Resin with attached FLAG-tagged protein was washed three times with Tris-buffered saline (TBS) pH 7.5 and resuspended in Laemmli loading buffer. Protein samples were separated by SDS-PAGE (sodium dodecyl sulfate polyacrylamide gel electrophoresis) and transferred onto nitrocellulose membranes. For DHX15-IP, 1.5 mg of FreeStyle HEK293 lysates was precleared with 10 µl protein A-coupled magnetic beads (Thermo Fisher Scientific) for 1 h at 4°C with gentle agitation. The precleared lysates were incubated with 1 µg of relevant antibody for 4 h, at 4°C with gentle shaking to allow the formation of immune complexes, followed by 2 h incubation with 20 µl of protein A magnetic beads to capture the complexes. Subsequent steps were the same as described in the case of FLAG-IP. The starting extract and immunoprecipitated proteins were examined by protein immunoblotting using either anti-FLAG (ANTI-FLAG^®^ M2 monoclonal, Sigma F3165) or the following primary antibodies: anti-CMTr1 (NB100-79786, Novus Biologicals), anti-DHX15 (NB100-586, Novus Biologicals).

### (d) Protein identification

For mass spectrometry (MS) identification, co-immunoprecipitated proteins were trypsin digested on anti-FLAG antibody-coated beads. The beads with bound protein complexes were resuspended in an equal volume of 100 mM ammonium bicarbonate. Cysteines were reduced in 10 mM DTT (30 min incubation at 56°C). After reduction, samples were alkylated in 50 mM iodoacetamide and incubated for 45 min in the dark, at room temperature. Trypsin digestion was performed with 500 ng of enzyme. After overnight incubation at 37°C, the supernatant was collected and beads were resuspended in 50% acetonitrile (ACN)/0.1% formic acid (FA). Elutions were combined and dried in a SpeedVac centrifugal evaporator. The sample was dissolved in 5% ACN/0.1% FA, prior to MS/MS (liquid chromatography-MS/MS) MS/MS analysis on an OrbiTrap (Thermo Fisher Scientific), performed in the Mass Spectrometry Laboratory of Institute of Biochemistry and Biophysics, Polish Academy of Sciences (IBB, PAS). Peptide ion identifications were ranked by Mascot (Matrix Science).

### (e) Overexpression and purification of recombinant proteins

For recombinant protein purification from eukaryotic expression systems, cells (High Five insect cells in the case of CMTr1 full-length and HEK293 FreeStyle in the case of CMTr1Δ135, the N-terminally truncated version of CMTr1) were resuspended in ice-cold lysis buffer and incubated with rotation for 1 h at 4°C. High Five cells were additionally subjected to ruption in a dounce homogenizer. Lysates were clarified by centrifugation for 30 min at 20 000***g*** and the resulting supernatant was incubated overnight with ANTI-FLAG^®^ M2 Affinity Gel (Sigma-Aldrich), with rotation at 4°C. Resin with attached FLAG-tagged protein was washed three times with TBS pH 7.5 and resuspended in an activity assay buffer. The affinity tag was removed by PreScission Protease (GE Healthcare) cleavage (4°C, overnight), followed by methyltransferase purification on a HiTrap™ heparin column. The protein was eluted as a single peak and frozen as aliquots at −80°C. The concentration of recombinant proteins was measured from SDS-PAGE by gel densitometry using ImageQuantTL Software (GE Healthcare). Analyses of ATP hydrolysis by CMTr1 protein preparations (from insect cells) in the presence of RNA revealed traces of ATPase activity, which could be caused, e.g., by a co-purified DHX15 protein homologue. This was not the case for CMTr1Δ135, which does not bind DHX15. The presence of helicase was taken into consideration during analyses of the results of CMTr1 activity assays, but it did not affect their interpretation.

Recombinant DHX15 overexpression in BL21DE3 cells was induced with 1 mM IPTG at an optical density of 0.8. Upon induction, cells were cultured for 12 h at 25°C. Protein was purified on HIS Select^®^ Nickel Affinity Gel (Sigma), in a buffer containing 20 mM HEPES pH 8, 300 mM NaCl, 10% (w/v) glycerol, 4 mM β-mercaptoethanol, 0.1% Triton X-100 and 10 mM imidazole. DHX15 was eluted by increasing imidazole concentration to 250 mM. DHX15-enriched fractions were pooled and further purified on a HiTrap Q column with 50 mM to 1 M gradient of NaCl in a buffer containing 20 mM HEPES pH 8, 5% (w/v) glycerol and 1 mM DTT. The protein observed as a single peak was collected, concentrated and frozen as aliquots at −80°C.

### (f) Preparation of RNA substrates

Substrates for methyltransferase assay were produced by *in vitro* transcription (T7 FlashScribe Kit, CellScript) in the presence of anti-reverse cap analogue (ARCA, Trilink), according to the manufacturer's recommendation. Templates for *in vitro* transcription were either PCR-amplified from appropriate templates or PCR-assembled by the Primerize technique [25]. All synthesized RNAs were gel purified by the ‘crush and soak’ method [26], precipitated and dissolved in water.

### (g) Methyltransferase assay

Methyltransferase activity assay was performed in BDHX buffer (20 mM Tris–HCl pH 8, 70 mM KCl, 3 mM MgCl_2_, 5 mM DTT, 100 µM ATP), optimized to maintain the activity of both methyltransferase and helicase enzymes, with the addition of 1 µCi (12.5 pmol) of [^3^H-methyl]-*S*-adenosyl methionine ([^3^H-methyl]-SAM) and 25 U of RiboLock nuclease inhibitor (Thermo Fisher Scientific). Reactions were carried out with 2.4 pmol of purified CMTr1 enzyme and 4.8 pmol of substrate RNA, in a total volume of 30 µl, at 37°C, for 1 h or the time indicated. Samples were applied on the Nylon membrane, dried and washed with 200 mM phosphate buffer (pH 6.5) to remove free [^3^H-methyl]-SAM. Then, samples were incubated for 30 min in scintillation liquid in the dark, and the measurements were performed by a scintillation counter (Tri-Carb 2900 TR Liquid Scintillation Analyzer, Packard Bioscience).

Methyltransferase assays in the presence of the helicase were performed in BDHX buffer at different CMTr1/DHX15 molar ratios (as indicated in figure legends). Mixtures containing both enzymes were incubated on ice for 10 min, before addition of the RNA substrate and SAM, and processed further as described above. Complex formation under such conditions was confirmed by His-DHX15 binding to CMTr1 followed by capturing the complexes on nickel-charged agarose beads (electronic supplementary material, figure S1).

### (h) Secondary structure prediction for RNA substrates used in biochemical analyses

RNA secondary structure, the minimum free energy (MFE), and the probability of individual base pairs were predicted using the ViennaRNA package [27], in particular by RNAfold (for single sequences) and RNAcofold (for a dimeric sequence), and by CentroidFold (v. 0.0.15) [28]. Design of RNA sequence variants with an altered strength of secondary structure was also guided by this approach.

### (i) Computational analysis of RNA secondary structure of 5′ ends of the human transcriptome

Sequences of human transcripts with accurately mapped 5′ ends, reported in [29], were obtained from the FANTOM consortium database—a collection of 5′ complete transcriptomes from five different databases (GENCODE release 19, Human BodyMap 2.0, miTranscriptome3, ENCODE2 and an RNA-seq assembly from 70 FANTOM5 samples). All these sequences were evaluated with TIEScore (Transcription Initiation Evidence Score), which determines the likelihood that a given transcript has a genuine transcription start site (TSS). Identifiers and coordinates of the sequences identified with this score as having the real TSS were obtained from a file, FANTOM_CAT.lv3_robust.bed, available at http://fantom.gsc.riken.jp/5/suppl/Hon_et_al_2016/data/assembly/lv3_robust/. For further analyses, we considered only 52 nt 5′-terminal fragments of transcripts, which corresponded in length to the size of the 5′-terminal hairpin structure present in the substrates used in this work. As a result, 279 252 non-identical 52 nt sequences (about 60% of the original dataset) were retained, which are hereafter referred to as the FANTOM dataset of 5′ ends (file FANTOM.bed.gz is available for download from ftp://genesilico.pl/iamb/data/CMTr1/).

Experimentally determined secondary structure of the human transcriptome was extracted from the Structure Surfer database [30], which was downloaded as a MySQL dump and used locally. Secondary structure assignments were retrieved and processed as described in the original article. For the final assignment of the secondary structure, if multiple experimental values were available, the highest value was used. Following the mapping of structure assignments on the FANTOM dataset of 5′ ends, 52 nt transcript fragments with more than 25% missing secondary structure data, or with constant experimental values, were excluded from further analysis. The final dataset used in this work consisted of 84 077 sequences, corresponding to 52 nt 5′ ends of transcripts with at least 39 residues (75%) characterized structurally as to their involvement in base-pairing or the lack thereof (file FANTOM_StructureSurfer.bed.gz is available for download from ftp://genesilico.pl/iamb/data/CMTr1/).

The KNIME data processing platform (v. 3.3.2) [31] and a set of in-house developed Python scripts were used for data handling, processing, and analysis.

## 3. Results

### (a) DHX15 interacts directly with the human cap1 methyltransferase CMTr1

To identify protein partners of human cap1 methyltransferase (CMTr1) that could regulate its activity, we performed immunoprecipitation experiments. FLAG-tagged CMTr1 was expressed in HEK293 cells (HEK293 Freestyle), and complexes were co-immunoprecipitated from the cell extracts with the use of anti-FLAG antibodies. Among all proteins co-immunoprecipitated with CMTr1, the highest ranked, according to Mascot score, was a 95 kDa protein, identified by MS as the ATP-dependent RNA helicase DHX15 (electronic supplementary material, table S1). The enrichment of DHX15 with FLAG-tagged CMTr1 was also confirmed by western blot analysis, using antibodies recognizing DHX15 (figure 1).

To determine whether the G-patch domain of CMTr1 mediates the interaction with DHX15, we performed co-immunoprecipitation (Co-IP) experiments with the N-terminally truncated version of CMTr1 (CMTr1Δ135). Results of the MS identification and western blot analysis demonstrated that the G-patch domain of CMTr1 is responsible for the interaction with DHX15 (figure 1*d*). The CMTr1–DHX15 interaction was further confirmed by a reciprocal Co-IP experiment using endogenous and FLAG-tagged DHX15 as bait (figure 1*b,c*). DHX15 co-immunoprecipitated endogenous CMTr1 and other G-patch-containing proteins previously known as DHX15 interactors (see electronic supplementary material, table S1). It has been shown previously that the OB-fold domain of DHX15 is responsible for interacting with G-patch-containing proteins [32]. However, we were unable to overexpress an OB-fold domain deletion variant of DHX15 suitable for Co-IP experiments. Hence, the specific interaction of this OB-fold domain with the G-patch of CMTr1 remains to be confirmed. DHX15 and CMTr1 protein variants analogous to those used in Co-IP experiments were prepared for subsequent *in vitro* studies, except for G-patch, which we were not able to purify as a homogeneous peptide (electronic supplementary material, figure S1).

### (b) DHX15 facilitates CMTr1 methyltransferase activity on RNA substrates with structured 5′ termini

**Figure 2.**
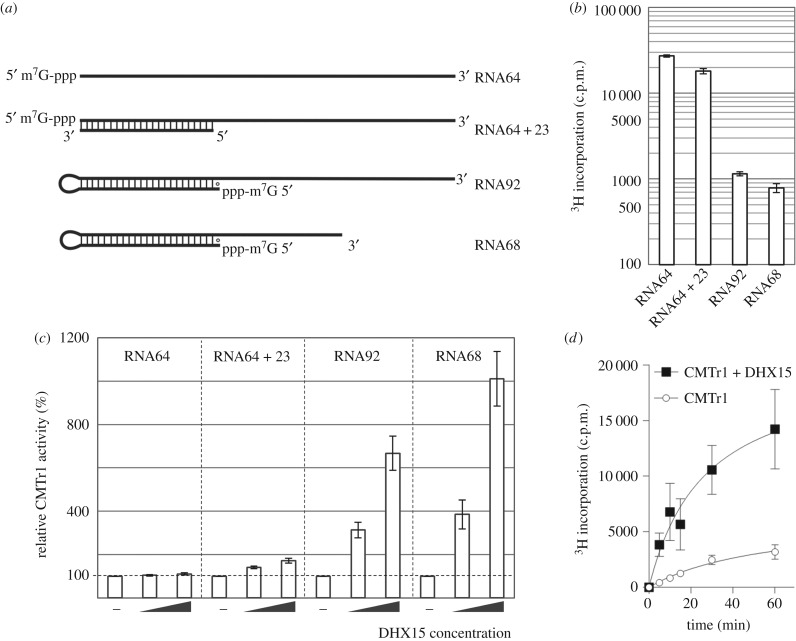
CMTr1 methyltransferase activity on RNA substrates with different types of secondary structure at 5′ ends. (*a*) Schematic of RNA substrates, with emphasis on the presence of a long base-paired region (other potential structures are not shown); canonical Watson–Crick base pairs (A-U and G-C) are indicated and wobble pairs G-U in the 5′-terminal hairpin are denoted as ‘○’. Sequences of RNA substrates and their predicted secondary structures are shown in electronic supplementary material, table S2 and figure S2. (*b*) CMTr1 methyltransferase activity, expressed as a level of tritium-labelled methyl group transfer onto different RNA substrates (shown in *a*). (*c*) Relative CMTr1 methyltransferase activity on different substrates in the absence or presence of DHX15, at 1 : 1 and 1 : 5 molar ratios. Data were normalized to CMTr1 activity on individual substrates as shown in (*b*) (set as 100% in each case) to illustrate the different relative effect of DHX15 on CMTr1 activity depending on the substrate. Electronic supplementary material, figure S3 shows the same data normalized to values obtained for RNA64. (*d*) The time-course of CMTr1 enzymatic activity on the RNA68 substrate in the presence (black squares) or absence (white circles) of DHX15 helicase (at 1 : 5 molar ratio). At the indicated time-points reactions were stopped by freezing in liquid nitrogen. In (*b–d*), results from three independent experiments are shown and error bars indicate standard deviations.

Based on the observation that DHX15 forms a complex with CMTr1 (figure 1), we hypothesized that DHX15 can influence the CMTr1 methyltransferase activity. Using the G-patch domain, CMTr1 could recruit DHX15 to the 5′ ends of CMTr1 substrates. DHX15 could, in turn, remove the secondary structure from base-paired 5′ regions, thereby enabling efficient methylation of the first transcribed nucleotide. We wondered if CMTr1 could preferentially methylate RNA molecules whose 5′-terminal residues are free from base-pairing. To test this hypothesis, we analysed CMTr1 methyltransferase activity on cap0-containing RNA substrates with different secondary structures at the 5′ terminus. First, the methyltransferase activity was tested on the RNA64 substrate used in our previous studies [10]. According to computational predictions with RNAfold, as well as other algorithms (see Methods for details), this substrate contained a very weak hairpin loop in the 5′ end and the MFE of the whole structure was relatively low, −15.30 kcal mol^−1^ (electronic supplementary material, table S2). Second, we developed two types of derivatives of the RNA64 substrate (figure 2*a*) to introduce a 23 bp double-stranded structure in their 5′ ends. First, RNA64 + 23 duplex comprised RNA64 and a 23 nt oligonucleotide fully complementary to the 5′ end of RNA64. According to RNAcofold, the dimeric RNA64 + 23 structure had predicted MFE of −47.80 kcal mol^−1^. Second, an RNA92 was constructed, where the 5′ end of RNA64 was extended by the addition of the aforementioned 23 nt sequence, connected by a GAAA tetranucleotide linker (electronic supplementary material, table S2 and figure S2) linker. At the 5′ end of RNA92, we included an additional G residue to enable efficient transcription by a T7 RNA polymerase. The resulting RNA folded into a stem–loop structure with 23 canonical base pairs and a wobble (G○U) pair formed by the extra G residue. According to RNAfold, the RNA92 structure had a predicted MFE of −49.10 kcal mol^−1^. We have also developed a series of RNA92 variants, in which the 3′ end was shortened by 24 residues: RNA68 and its derivatives (electronic supplementary material, table S2). The secondary structures of CMTr1 substrates (RNA64, RNA64 + 23 and RNA68 derivatives) were verified by the SHAPE method (see electronic supplementary material, figure S2 for details). The experimentally derived SHAPE reactivity profiles were found to support the predicted secondary structures of our RNA substrates, and the structures presented in this work are obtained based on SHAPE-assisted predictions (electronic supplementary material, figure S2).

Compared with the original RNA64 substrate, which had a relatively weakly structured 5′ end, the methylation of the bi-molecular RNA64 + 23 substrate was reduced by approximately 30%. However, the methylation of the single-stranded RNA92 substrate containing a hairpin at the 5′ end was strongly (approx. 20-fold) diminished compared with the original RNA64 substrate. Likewise, an approximately 30-fold decreased cap1 methylation was observed for the RNA68 substrate, which was used in further experiments (figure 2*b*).

For comparison, we measured the methyltransferase activity of CMTr1 towards the aforementioned substrates in the presence of DHX15. DHX15 did not exhibit cap1 methyltransferase activity independently of CMTr1; however, upon addition of DHX15, CMTr1 methylated RNA more efficiently (figure 2*c*; electronic supplementary material, figure S3). This effect is attributed to the 2′-*O*-ribose methyltransferase specificity of CMTr1 as shown in electronic supplementary material, figure S4. The stimulatory effect of DHX15 on CMTr1 activity was minimal for RNA64, which has a relatively weakly structured 5′ end, and it was very strong for RNA92 and RNA68 substrates, in which the 5′ end is highly structured, and in which the first transcribed nucleotide is involved in base-pairing (figure 2; electronic supplementary material, figure S5). Augmenting the concentration of DHX15 increased cap1 methylation in a dose-dependent manner. Adding equimolar concentrations of CMTr1 and DHX15 resulted in over threefold increase in the methyltransferase activity towards RNA92 and RNA68 substrates (those with a hairpin at the 5′ end), in comparison with CMTr1 alone. Moreover, the time-course measurements of CMTr1 methyltransferase activity revealed that upon adding a pre-formed CMTr1–DHX15 complex to the reaction, the substrate was methylated more efficiently compared with CMTr1 alone (figure 2*d*). Thus, the formation of the CMTr1–DHX15 complex appears to be important for the effective methylation of substrates containing the base-paired 5′ regions.

**Figure 3.**
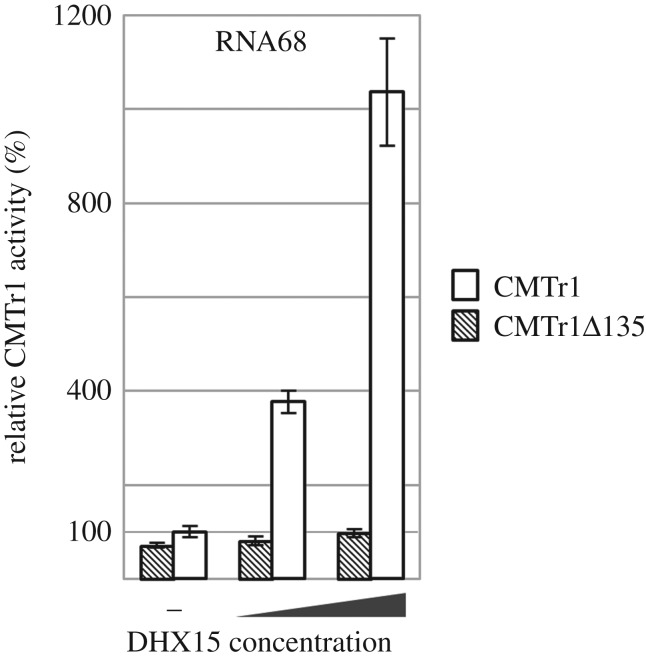
CMTr1 deletion mutant (CMTr1Δ135) activity towards the capped RNA68 substrate in comparison with the full-length protein (CMTr1) in the presence or absence of DHX15 helicase at 1 : 1 and 1 : 5 ratios. Data were normalized to CMTr1 activity. Results from three independent experiments are shown and error bars indicate standard deviations.

To analyse the role of the direct interaction between CMTr1 and DHX15, we tested the impact of DHX15 on the cap1 methyltransferase activity of a CMTr1 deletion mutant lacking the G-patch domain (CMTr1Δ135) responsible for the interaction. Similarly to the full-length CMTr1, CMTr1Δ135 methylated efficiently the RNA64 substrate (electronic supplementary material, figure S6), whereas its activity towards RNA68 was lower. However, in contrast to the activity of full-length CMTr1, DHX15 had negligible influence on methylation of RNA68 by CMTr1Δ135. This result strongly suggests that direct interaction of CMTr1 and DHX15, mediated by the G-patch domain of CMTr1, is required for the stimulation of CMTr1 methyltransferase activity on RNA with a highly structured 5′ end (figure 3).

### (c) Stimulation of CMTr1 methyltransferase activity by DHX15 depends on the strength of the secondary structure in the 5′ terminus of the RNA substrate

**Figure 4.**
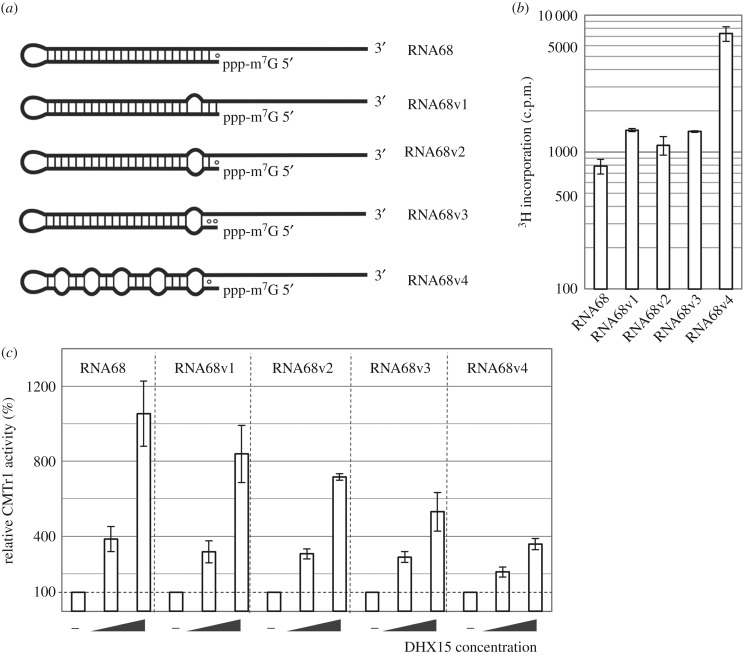
Influence of RNA secondary structure on DHX15-stimulated CMTr1 methyltransferase activity. (*a*) Schematic of RNA substrates with different base-pairing strength at their 5′ ends used for CMTr1 activity assay. Emphasis is on the presence of a long base-paired region in the 5′ terminus (other potential structures are not shown); canonical Watson–Crick base pairs (A-U and G-C) are indicated and ‘○’ denotes wobble pairs G-U. Sequences of RNA substrates and their predicted secondary structures are shown in electronic supplementary material, table S2 and figure S2. (*b*) Activity of CMTr1 towards individual substrates in the absence of DHX15. (*c*) CMTr1 activity in the presence of DHX15 at 1 : 1 and 1 : 5 molar ratio. Data were normalized to CMTr1 activity on individual substrates as shown in (*b*) (set as 100% in each case) to illustrate the difference in relative effect of DHX15 on CMTr1 activity depending on the substrate. In (*b*,*c*), results from three independent experiments are shown and error bars indicate standard deviations.

To further elucidate the influence of RNA secondary structure on the CMTr1 activity we prepared four derivatives of the RNA68 substrate that differ in the strength of secondary structure in the 5′-terminal region (figure 4*a*). Changes introduced in the RNA68 sequence ranged from single nucleotide substitutions that interfered with the ability of the very first 5′-terminal residues to form base pairs, to a series of substitutions that weakened the entire hairpin present in the original RNA68 substrate (electronic supplementary material, figure S2). The results of *in vitro* methylation assays show that RNAs with a relatively stable secondary structure at their 5′ ends were poorly methylated by CMTr1 alone (figure 4*b*). Importantly, while the liberation of the target residue (the first nucleotide of the transcript) from base-pairing has a relatively mild positive effect on methylation efficiency by CMTr1, the disruption of a long secondary structure has a strong effect. Thus, the presence of a relatively long helix in the 5′ end of the substrate has a strong inhibitory effect on CMTr1 activity, even if the target residue is unpaired. Upon addition of DHX15 to the methylation reaction, RNA68(v1–v4) substrates were methylated more efficiently (figure 4*c*; electronic supplementary material, figure S7). The effect of helicase activity of DHX15 was consistent with the strength of the secondary structure, which has to be unwound for the CMTr1 methylation to access the 5′-terminal residues in a single-stranded conformation. The regulatory effect of DHX15 on CMTr1 activity was stronger on RNA substrates with a long uninterrupted helix. However, it was also clearly seen on the RNA68v4 substrate, in which the series of canonical base pairs in the helix was interrupted by multiple mismatches.

### (d) CMTr1 does not stimulate DHX15 strand displacement activity

DHX15 helps CMTr1 in methylation of structured RNA substrates. Hence we wanted to check if the reciprocal effect takes place. We found that the ATPase activity of DHX15 was stimulated by CMTr1, but not by the CMTr1Δ135 variant (electronic supplementary material, figure S8), regardless of the presence of RNA (data not shown), which provides further evidence for direct interactions between these two proteins mediated by the G-patch domain of CMTr1. Firstly, we showed that DHX15 exhibited the strand displacement activity towards a bi-molecular double-stranded RNA (RNA40 + 23) and its variants, which had essentially the same sequence and structure as the single-stranded RNA68 variants, with the GAAA loop replaced by a blunt end (electronic supplementary material, table S2 and figure S9). Secondly, we tested the influence of CMTr1 on the strand displacement activity of DHX15. These tests were carried out on the uncapped RNA40 + 23 duplex as well as on the RNA64 + 23 duplex, in which RNA64 was 5′-capped (electronic supplementary material, figure S10). In none of the cases, did we observe any increase in the strand displacement activity due to the addition of CMTr1 to DHX15 (representative results are shown in electronic supplementary material, figure S10). Negligible strand displacement was observed due to CMTr1 alone, which explains its poor activity on RNAs with highly structured 5′ ends. Interestingly, we observed inhibition of strand displacement activity of DHX15 by CMTr1Δ135 on the capped RNA64 + 23 substrate and, to a lesser extent, on the uncapped RNA40 + 23 substrate. The inhibitory effect of CMTr1Δ135 may be due to its binding to the RNA substrate (partly specific to the cap, partly nonspecific), while being unable to interact with DHX15, which may result in blocking DHX15 from exerting its strand displacement activity. This hypothesis requires further investigation including structural studies.

### (e) A significant fraction of CMTr1 substrates have structured 5′-terminal regions

To assess the potential biological relevance of the identified functional interaction between CMTr1 and DHX15, we analysed the prevalence of secondary structure at 5′ ends of human RNAs that are potential substrates for CMTr1. We focused the study on 5′-terminal regions of human transcripts with accurately mapped 5′ ends [29] and selected 52 nt segments of these RNAs, which corresponded in length to the 5′-terminal stem–loop structure present in the RNA substrates analysed biochemically in this work. For these segments, we mapped the available experimental data on *in vivo* secondary structure determination, as deposited in the Structure Surfer database [30]. Ultimately, we analysed 84 077 unique 52 nt sequences corresponding to 5′ ends of transcripts, in which at least 75% of positions had experimental information available as to their involvement in base-pairing or the lack thereof (see Methods for details). We found that a substantial number of transcripts had relatively high content of base-paired residues in the 52 nt 5′-terminal regions analysed: 55.62% of transcripts had at least 50% of residues base-paired, and 11.94% had at least 75% of the residues base-paired. For comparison, in RNA68v4, 69% of residues in the 5′-proximal 52 nt region are base-paired. Furthermore, as many as 24.38% of sequences in the dataset analysed had the first ten residues fully base-paired. While these transcripts are expected substrates for CMTr1, they possess *in vivo* secondary structure that can be an obstacle for CMTr1 activity but can be potentially unwound by DHX15.

## 4. Discussion

In higher eukaryotes, 5′ ends of all mRNAs and many non-coding RNAs (including long non-coding RNAs (lncRNAs), small nucleolar RNAs (snoRNAs), and the majority of small nuclear RNAs (snRNAs)) are modified by ribose 2′-*O*-methylation on the first transcribed nucleotide. This additional methylation enhances translation of mRNA during oocyte maturation [33,34] and facilitates splicing of small nuclear RNAs [35]. Beyond these functions, the cap1 structure is essential for the non-self discrimination of innate immune response against foreign RNA [36].

Recently, we have determined the crystal structures of the active CMTr1 catalytic domain in complex with a methyl group donor SAM and a capped 4 nt ribonucleotide (m^7^GpppGAUC), thereby revealing the mechanism of CMTr1 interactions with the 5′ terminus of a capped RNA [15]. Analysis of this structure shows the lack of contacts between the bases of the transcript (GAUC) and the CMTr1 protein, which suggests that substrate binding and methylation are sequence-independent. In the crystal structure, the four 5′-terminal residues of the RNA substrate assume a stacked conformation, similar to that in the double-stranded A-form RNA. Superposition of these residues onto an idealized dsRNA helix suggests that the first three residues (GAU), including the residue methylated by CMTr1, could in principle form base pairs with complementary residues, without interfering with RNA binding by the enzyme. However, the potential binding partner of the fourth residue and the following six base pairs would generate extensive steric conflicts with the protein (electronic supplementary material, figure S11). Hence, the presence of a double-stranded region in the 5′ terminus of RNA is structurally incompatible with its binding and methylation by CMTr1. As a consequence, the extent to which the ten or so nucleotide residues at the 5′ terminus of a transcript are involved in base-pairing may affect the structural compatibility of that substrate with the RNA-binding pocket of CMTr1 and hence affect the efficiency of its methylation.

In this work, we found that CMTr1 indeed acts poorly on RNA substrates with an extensive secondary structure in the 5′ terminus, e.g. RNA68 and RNA92. In order to methylate these targets efficiently, CMTr1 may require the secondary structure to be removed, which could be a task for an RNA helicase. We also found that the activity of CMTr1 varied strongly between RNAs in which the 5′ terminus was base-paired to a short complementary oligo, and RNAs in which the base-pairing was intra-molecular. This could be attributed to several factors, including RNA sequence, the ability of CMTr1 to displace the small oligonucleotide, the cooperative effect of having the duplex strands covalently linked (and thus stronger RNA duplex stability), and interactions of CMTr1 with the 3′-terminal part of the substrate (beyond the base-paired region). In our experiments, CMTr1 did not show any strand displacement activity on its own (electronic supplementary material, figure S10) and we saw no evidence for sequence specificity (data not shown). We suspect that the RNA structure plays the strongest role, including the possibility that the 3′-terminal part of the substrate may contribute to steric interference if the folding of the 5′-terminal part brings the rest of the molecule too close to CMTr1.

In line with the potential requirement for help in removal of the secondary structure from the 5′ end of the RNA, we found that CMTr1 interacts strongly with an ATP-dependent RNA helicase DHX15. Thus far, DHX15 and its yeast homologue Prp43 have been implicated in diverse cellular functions involving RNA metabolism, including splicing and ribosome biogenesis, but not in RNA modification. It is known that DHX15/Prp43 distribution between individual target pathways is regulated by its interplay with the various G-patch protein cofactors [37]. DHX15/Prp43 is recruited and activated in splicing by Ntr1 and TFIP11 proteins, respectively. Stimulation of Prp43 helicase activity by Ntr1 splicing factor in yeast is required for lariat–intron release, and the deletion of its human homologue, TFIP11, impairs spliceosome disassembly by DHX15 [22,38]. The G-patch protein RBM5 is a known regulator of alternative splicing in apoptosis. RBM5 is able to activate helicase and ATPase activity of DHX15 and it is hypothesized that it can regulate splicing owing to this ability [23]. On the other hand, the G-patch protein PINX1 in humans, and Pfa1 and Gno1 in yeast are known to stimulate Prp43/DHX15 activity in ribosome biogenesis [24,39]. Recently, it has been shown that the G-patch protein NF-κB-repressing factor (NKRF) forms a pre-ribosomal subcomplex with DHX15 and with the 5′–3′ exonuclease XRN2, which is essential for processing of pre-rRNAs and the turnover of excised spacer fragments [20]. DHX15 has also been implicated in the immune response to viral infection [40].

CMTr1 is a G-patch protein, and we found that it binds DHX15. Co-IP experiments showed that a CMTr1 variant that lacks the G-patch domain does not bind DHX15, and it also cannot be aided by DHX15 in the methylation of RNAs containing strongly structured 5′ termini. This provides evidence of G-patch-dependent interaction between CMTr1 and DHX15, which enables CMTr1 to methylate efficiently all types of capped substrates, regardless of their secondary structure. The mechanistic details of this process remain to be elucidated.

Based on the available data, we speculate that CMTr1 and DHX15 form a relatively stable complex, using the G-patch domain of CMTr1 and the OB-fold domain of DHX15 (electronic supplementary material, figure S1B), which can bind capped RNAs. The CMTr1 can either directly methylate the first transcribed nucleotide in RNAs with unstructured 5′ termini, or use the help of a physically associated DHX15 protein to free the 5′-terminal region from base-pairing, thereby making it available for methylation (electronic supplementary material, figure S11C). We were unable to see a stimulatory effect of CMTr1 on the DHX15 strand displacement activity with any of the RNA substrates used. On the other hand, the decoupling of physical interactions between the enzymes by deletion of the G-patch domain of CMTr1 leads to inhibition of that activity. Additional studies, in particular, structure determination for the CMTr1–DHX15 complex with a substrate RNA and detailed biochemical analyses of kinetic parameters, are necessary to understand the interplay of these enzymes.

RNA methylation aided by an RNA helicase has been reported in the course of rRNA modification mediated by snoRNAs during ribosome production in human cells [41]. It was found that the action of several late-acting snoRNAs requires the activity of DDX21 helicase at the methylation target sites in pre-rRNAs, which must base-pair with the affected snoRNAs for the modification to occur [42,43]. A similar mechanism of action in connection with some modification-guiding snoRNAs was suggested for Rok1 helicase in yeast [41].

The requirement for RNA unwinding activity to enable methylation of highly structured substrates has also been reported for RlmKL methyltransferase in *E. coli* [44]. This enzyme comprises two fused methyltransferase domains, which introduce two different base modifications in helix 74 of *E. coli* 23S rRNA, namely m^7^G2069 and m^2^G2445. Both domains of RlmKL cannot bind helix 74 at the same time in the folded structure of the 23S rRNA, and the RlmL domain preferentially recognizes and methylates the single-stranded substrate rather than the duplex substrate, suggesting that helix 74 should be unwound to serve as a substrate for m^2^G2445 methylation. In this case, an RNA unwinding activity was found to be associated directly with RlmKL and *in vitro* assays have not indicated any requirement for the additional helicase activity [44].

While this work was under review, another study reported CMTr1–DHX15 interactions [45]. The authors also found CMTr1 and DHX15 to interact directly. However, they reported an opposite effect of DHX15 presence on CMTr1 activity. In that study, the human CMTr1 protein was purified from bacterial cells, which could explain its different properties. It will be interesting to determine the activity of bacterially expressed CMTr1 on RNA substrates with different structures.

Thus far, RNA methylation has been found to influence RNA structure. In particular, m^6^A alters local RNA structure and acts as a dynamic switch that can control the RNA-structure-dependent accessibility of RNA binding motifs [46,47]. 2′-*O*-Methylation was also suggested to affect RNA folding and structural stability [48]. Here, we demonstrate a reverse functional relationship, in which the RNA secondary structure determines its own potential to be methylated. The discovery of DHX15 involvement in RNA methylation suggests that the CMTr1 activity on structured substrates can be regulated to enable efficient modification. Thus, our results suggest a new link between RNA epigenomics, RNA structure and RNA helicases.

## Supplementary Material

Supplementary Tables and Figures

## Data accessibility

Tables and figures available as part of the electronic supplementary material. Additional files with transcript datasets are available for download at ftp://genesilico.pl/iamb/data/CMTr1/.
