# Supplementary material for: Human RNA cap1 methyltransferase CMTr1 cooperates with RNA helicase DHX15 to modify RNAs with highly structured 5′ termini
Source: Philos Trans R Soc Lond B Biol Sci. 2018 Nov 5;373(1762):20180161. doi: 10.1098/rstb.2018.0161 (PMC6232587; doi:10.1098/rstb.2018.0161)
Supplement: Supplementary Tables and Figures [file rstb20180161supp1.docx]

**SUPPLEMENTARY INFORMATION**

**Human RNA cap1 methyltransferase CMTr1 cooperates with RNA helicase DHX15**

**to modify RNAs with highly structured 5′ termini**

Diana Toczydlowska-Socha^1,#^, Magdalena Zielinska^1,#^, Malgorzata Kurkowska^1,#^, Astha^1^, Catarina F. Almeida^1^, Filip Stefaniak^1^, Elzbieta Purta^1,*^, Janusz M. Bujnicki^1,2,*^

^1^ Laboratory of Bioinformatics and Protein Engineering, International Institute of Molecular and Cell Biology, ul. Trojdena 4, 02-109 Warsaw, Poland

^2^ Institute of Molecular Biology and Biotechnology, Faculty of Biology, Adam Mickiewicz University, ul. Umultowska 89, 61-614 Poznan, Poland

^#^ D.T.S., M.Z. and M.K. contributed equally and should be considered Joint First Authors

^*^ To whom correspondence should be addressed. Email: [iamb@genesilico.pl.](mailto:iamb@genesilico.pl) Correspondence may be also addressed to Elzbieta Purta, Email: [ela@genesilico.pl](mailto:ela@genesilico.pl)

This Supplementary Information contains:

Supplementary Figures S1–S11

Supplementary Tables S1-S2

**SUPPLEMENTARY DATA**

**Supplementary Table 1.** Proteins identified by mass spectrometry as CMTr1 or DHX15 interactors.

**
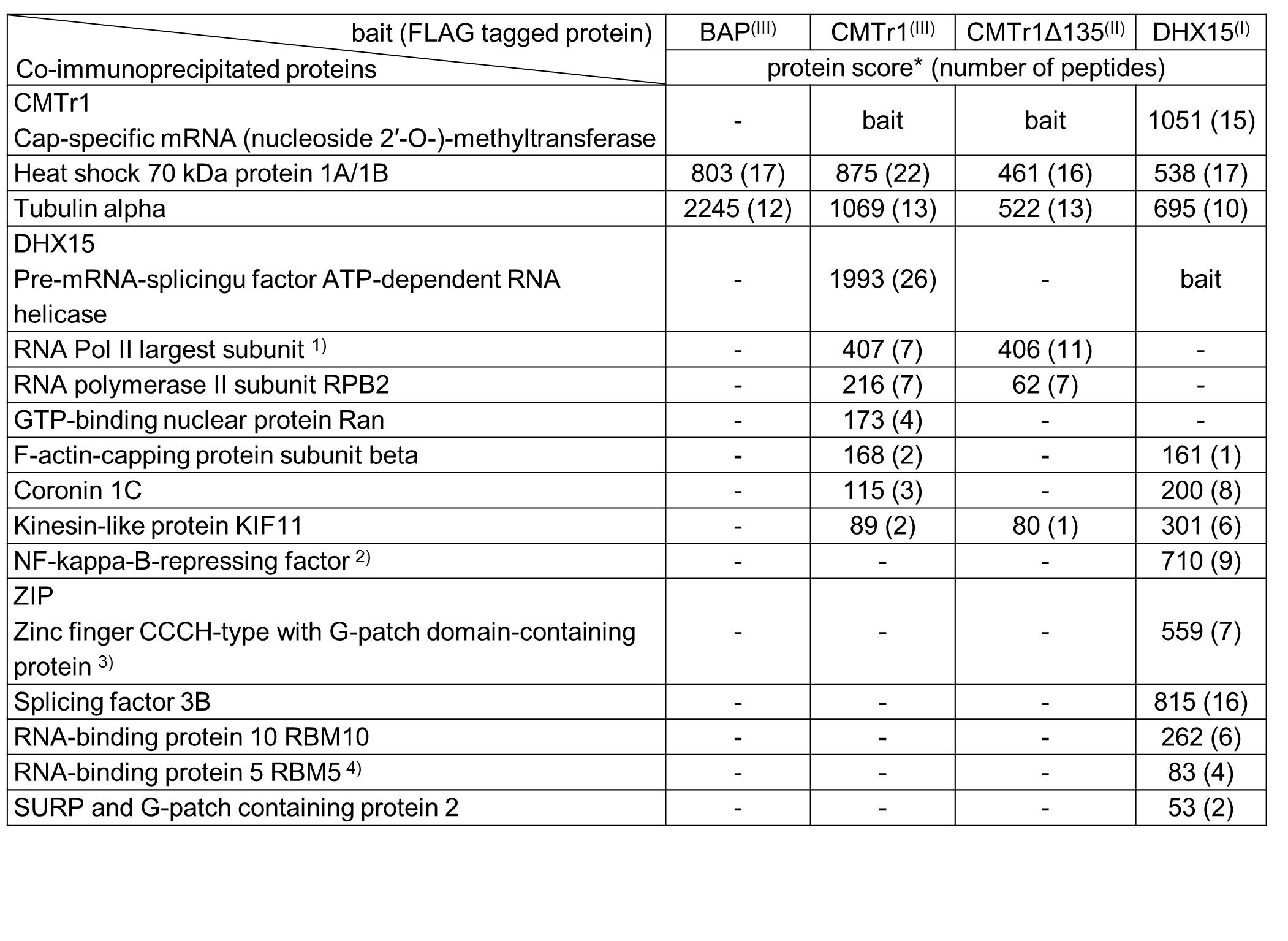
**Mass spectrometry identification of CMTr1-DHX15 binding proteins from HEK293 cells. The identified proteins are arranged by protein score, which reflects the number of unique peptides found for each protein and their relative abundance, as well as the percentage of sequence coverage. Non-specific binders were filtered-out by comparison with the control sample (bait – FLAG-tagged bacterial alkaline phosphatase protein). (I), (II), (III) – numbers of Co-IP experiments. * Protein scores for representative experiments are shown. Proteins previously reported to interact with CMTr1 [1] or DHX15 [2, 3, 4].

**Supplementary Table 2:** Sequences of RNA substrates used in the study.

| **RNA** | **Sequence (5ʹ to 3ʹ)** | **MFE of the whole structure [kcal/mol ]** | **stability of the 5ʹ-terminal structure [kcal/mol ]** |
| --- | --- | --- | --- |
| RNA64 | GGGUAACGCUAUUAUUACAAAGCUCUUUUAUGUAGUGUGCGUACCACGGUAGCAGGUACUGCGC | -15.30 | -9.50 |
| RNA64+23 | GGGUAACGCUAUUAUUACAAAGCUCUUUUAUGUAGUGUGCGUACCACGGUAGCAGGUACUGCGC + GCUUUGUAAUAAUAGCGUUACCC | -47.80 | -36.50 |
| RNA92 | GGCUUUGUAAUAAUAGCGUUACCCGAAAGGGUAACGCUAUUAUUACAAAGCUCUUUUAUGUAGUGUGCGUACCACGGUAGCAGGUACUGCGC | -49.10 | -38.10 |
| RNA68 | GGCUUUGUAAUAAUAGCGUUACCCGAAAGGGUAACGCUAUUAUUACAAAGCUCUUUUAUGUAGUGUGC | -39.00 | -38.10 |
| RNA68v1 | GGCUUUGUAAUAAUAGCGUUACCCGAAAGGGUAACGCUAUUAUUACAAAGGUCUUUUAUGUAGUGUGC | -35.30 | -34.50 |
| RNA68v2 | GGCUUUGUAAUAAUAGCGUUACCCGAAAGGGUAACGCUAUUAUUACAAUGCUCUUUUAUGUAGUGUGC | -35.20 | -34.30 |
| RNA68v3 | GGCUUUGUAAUAAUAGCGUUACCCGAAAGGGUAACGCUAUUAUUACAAUGUUCUUUUAUGUAGUGUGC | -32.80 | -32.00 |
| RNA68v4 | GGCUUUGUAAUAAUAGCGUUACCCGAAAGGCUAAGGCUCUUAUCACAAUGUUCUUUUAUGUAGUGUGC | -14.50 | -14.10 |
| RNA40 | GGGUAACGCUAUUAUUACAAAGCUCUUUUAUGUAGUGUGC + GCUUUGUAAUAAUAGCGUUACCC | -37.70 | -36.50 |
| RNA40v1 | GGGUAACGCUAUUAUUACAAAGGUCUUUUAUGUAGUGUGC + GCUUUGUAAUAAUAGCGUUACCC | -34.50 | -34.50 |
| RNA40v2 | GGGUAACGCUAUUAUUACAAUGCUCUUUUAUGUAGUGUGC + GCUUUGUAAUAAUAGCGUUACCC | -33.90 | -32.70 |
| RNA40v3 | GGGUAACGCUAUUAUUACAAUGUUCUUUUAUGUAGUGUGC + GCUUUGUAAUAAUAGCGUUACCC | -31.40 | -31.30 |
| RNA40v4 | GGCUAAGGCUCUUAUCACAAUGUUCUUUUAUGUAGUGUGC + GCUUUGUAAUAAUAGCGUUACCC | -13.50 | -13.50 |

Minimum free energies (MFEs) of RNA substrates were calculated with RNAfold software for single-stranded RNAs and RNAcofold for bi-molecular RNAs. Sequences of 5′ terminal structures are underlined.


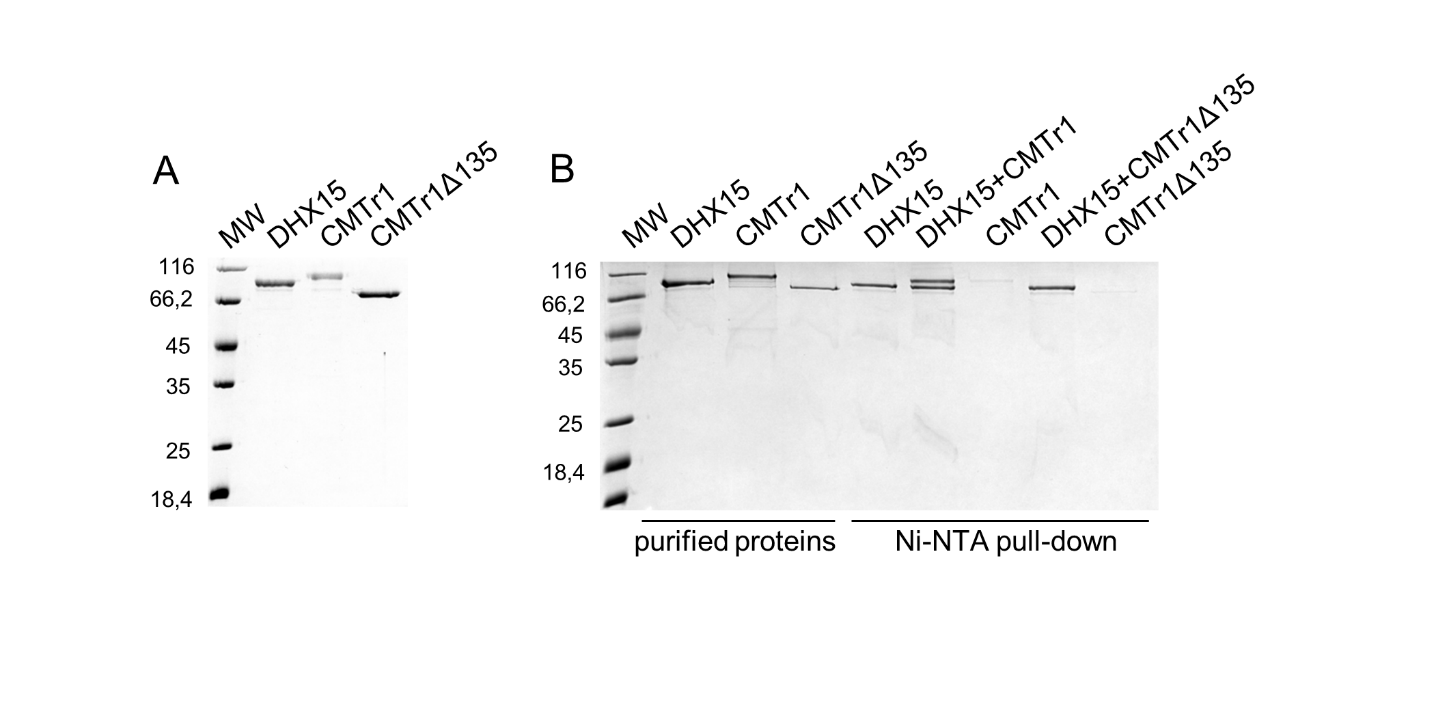


**Supplementary Figure 1.** **SDS-PAGE analysis of recombinant proteins and their complexes used in vitro assays.** A) Size and purity of each recombinant protein were evaluated using Coomassie blue stained, 12% SDS-PAGE gel, containing 0.1% SDS, under reducing conditions. See Materials and Methods for details of protein purification procedures. B) SDS-PAGE analysis of DHX15-CMTr1 complexes. CMTr1 or its variant were incubated with His-tagged DHX15 bound to nickel-charged agarose beads for 10 minutes, on ice in BDHX buffer. The beads were washed 3 times with TBS buffer to remove unbound proteins. The complexes were eluted with TBS buffer supplemented with 250 mM imidazole. DHX15, CMTr1 and CMTr1Δ135 alone were used as controls. Proteins and/or their complexes were resolved on 12% SDS-PAGE gel.

**
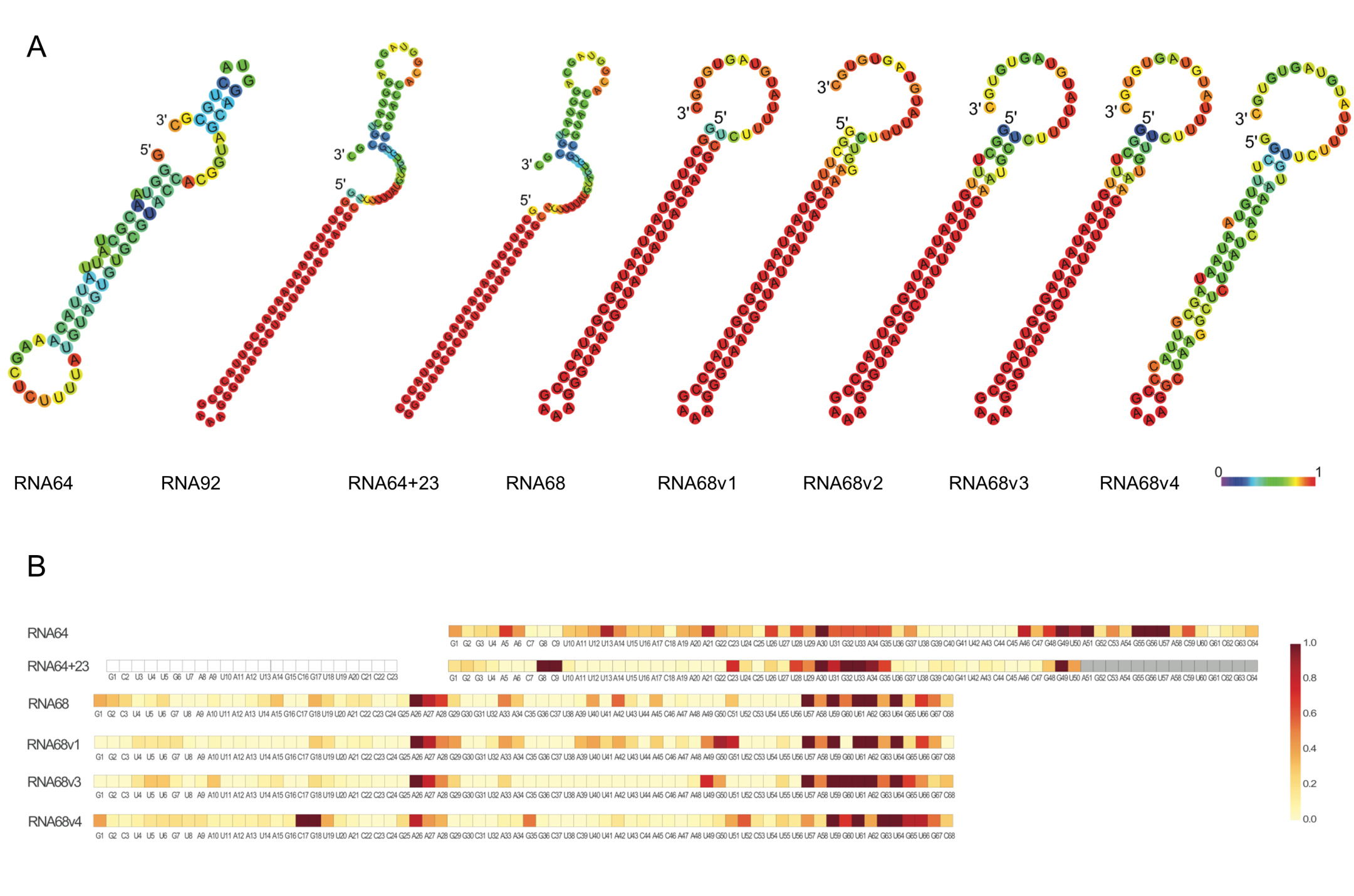
**

**Supplementary Figure 2: Analysis of secondary structures of RNA substrates.** A) Secondary structures predicted for RNA substrates analyzed in this work. For RNA64, RNA64+23, RNA68, RNA68v1, RNA68v3, and RNA68v4 the structures were predicted with the use of SHAPE experiments (shown in panel B). Individual residues are colored by base-pairing probabilities, according to CentroidFold. For unpaired regions, the color denotes the probability of being unpaired. CentroidFold does not generate models for bi-molecular RNAs. Consequently, the model of the RNA64+23 structure is based on the structure of RNA92 using secondary structure prediction from RNAcofold, which enables analysis of bi-molecular RNAs, but does not generate graphical output itself. Minimum free energies (MFEs) of the shown structures are listed in Supplementary Table 2. B) Heat maps showing the results of secondary structure probing by SHAPE (selective 2′-hydroxyl acylation analyzed by primer extension). The experiment was done as described previously [5]. For positive reaction, 2 pmoles of RNA, after folding in the buffer, were treated with 1 µL of 100 mM NMIA while the control reactions were incubated with 1 µL DMSO. In order to reverse transcribe (+/-) NMIA reactions, 0.48 µL of 5 mM VIC labeled primer was used. Dideoxy sequencing ladders were generated by reverse transcription of unmodified RNA with the use of 6-FAM labeled primer in the presence of ddCTP or ddTTP [6]. The fluorescently labeled cDNAs were resolved on an Applied Biosystems 3130 capillary electrophoresis instrument, and the raw capillary electrophoresis traces were processed using QuSHAPE software as described in [7]. Integrated intensities were normalized by dividing the data set by the average of the 8% most reactive nucleotides, after first excluding the top 2% reactivities. With this normalization, the mean cleavage intensity of the most highly reactive nucleotides becomes 1.0. The normalized reactivity of nucleotide below 0.3 shows that the nucleotide is constrained; greater than 0.3 and below 0.7 shows that the nucleotide is likely to be single-stranded and greater than 0.7 shows that the nucleotide is single-stranded [8].


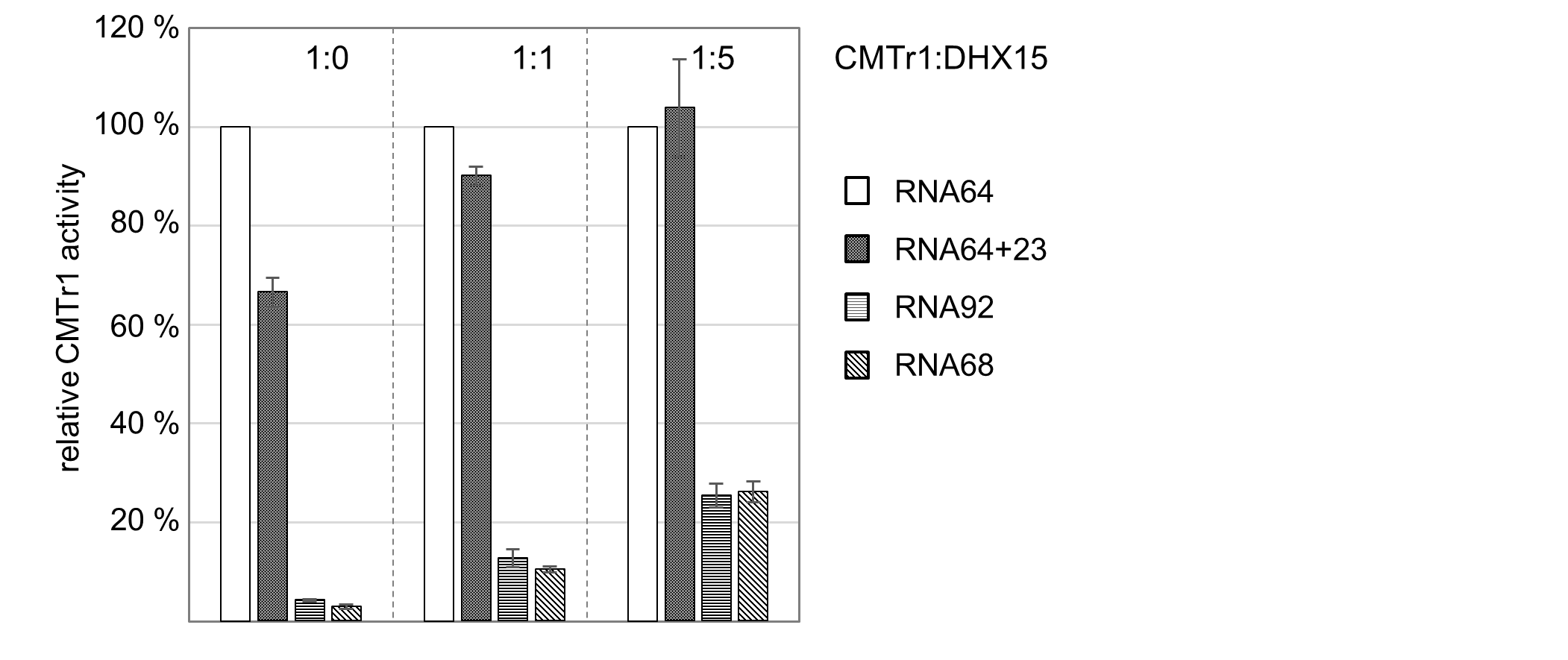


**Supplementary Figure 3.** **CMTr1 methyltransferase activity towards different RNA substrates normalized to values observed for RNA64 (shown as 100%).** This series of plots illustrates the same data as shown in Figure 2D. This comparison shows the difference in the CMTr1 effectiveness to methylate different substrates and emphasizes the scale of influence of DHX15 on the CMTr1 activity, at 1:1 and 1:5 molar ratios. With increasing concentrations of DHX15, the relative CMTr1 activity on RNA64+23, RNA92, and RNA68 increases.


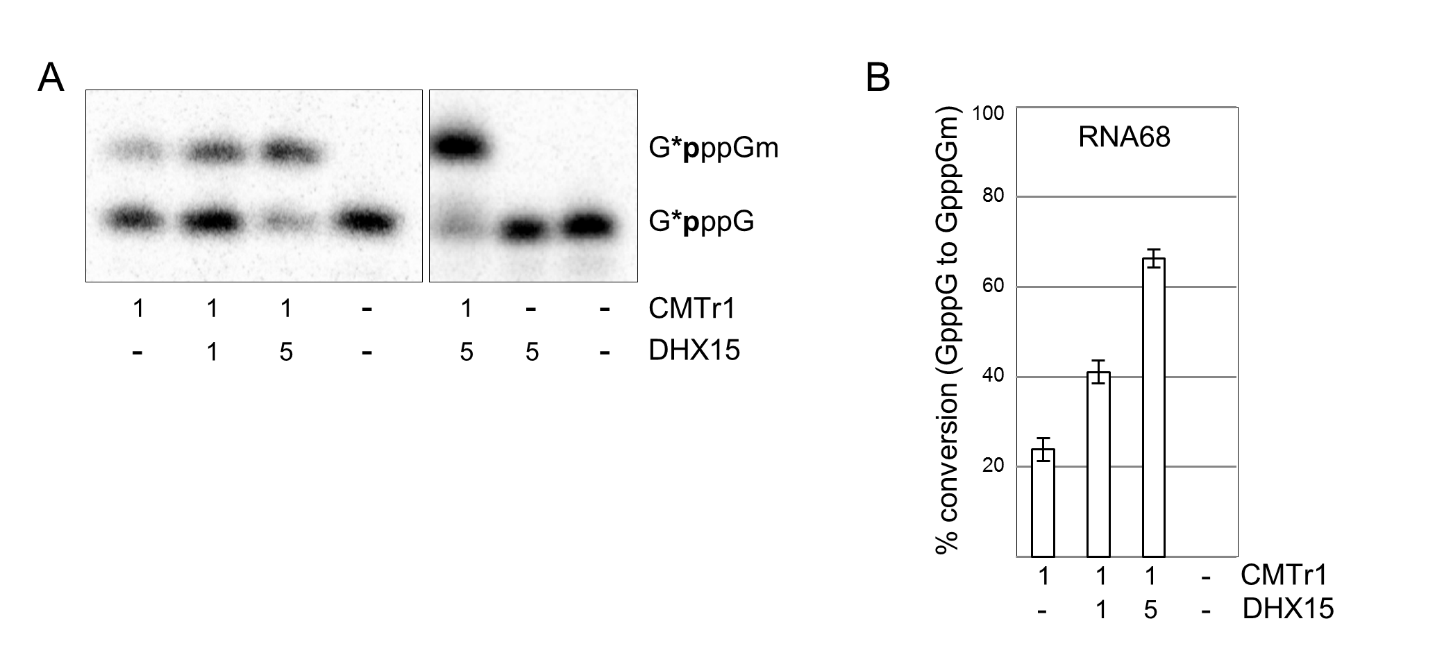


**Supplementary Figure 4.** C**MTr1 methyltransferase activity towards the first transcribed nucleotide of capped RNA68.** RNA molecules were subjected to capping reactions using vaccinia virus capping enzyme (VCE) ScriptCap (Epicentre). Reactions were carried out following the manufacturer recommendations with the addition of 10 μCi [α-^32^P] GTP (3000 Ci/mmol; Hartman Analytic GmbH) to form GpppN- (capG) structure. Following the reaction, ^32^P-labeled capped RNA molecules were purified by extraction with phenol/chloroform and precipitated with ethanol. The RNA substrate was subjected to methyltransferase reaction (as described in Materials and Methods) with 250 μM non-radiolabeled SAM. Following the methylation reaction, the RNA was digested with 1 U nuclease P1 (Sigma) in 10 mM Tris–HCl (pH 7.5), 10 mM MgCl_2_ and 50 mM NaCl for 1 hour at 60°C. The digestion products were resolved on a 21% polyacrylamide/8 M urea gel and visualized by PhosphorImaging (Typhoon Trio, GE Healthcare). A) A representative autoradiogram showing the results of the experiment described above. G*pppG-RNA68 was treated by CMTr1 and DHX15 at 1:1 and 1:5 molar ratios, respectively. The asterisk indicates the ^32^P-labeled phosphate group. B) Quantification of results of three independent experiments. Error bars indicate standard deviations.


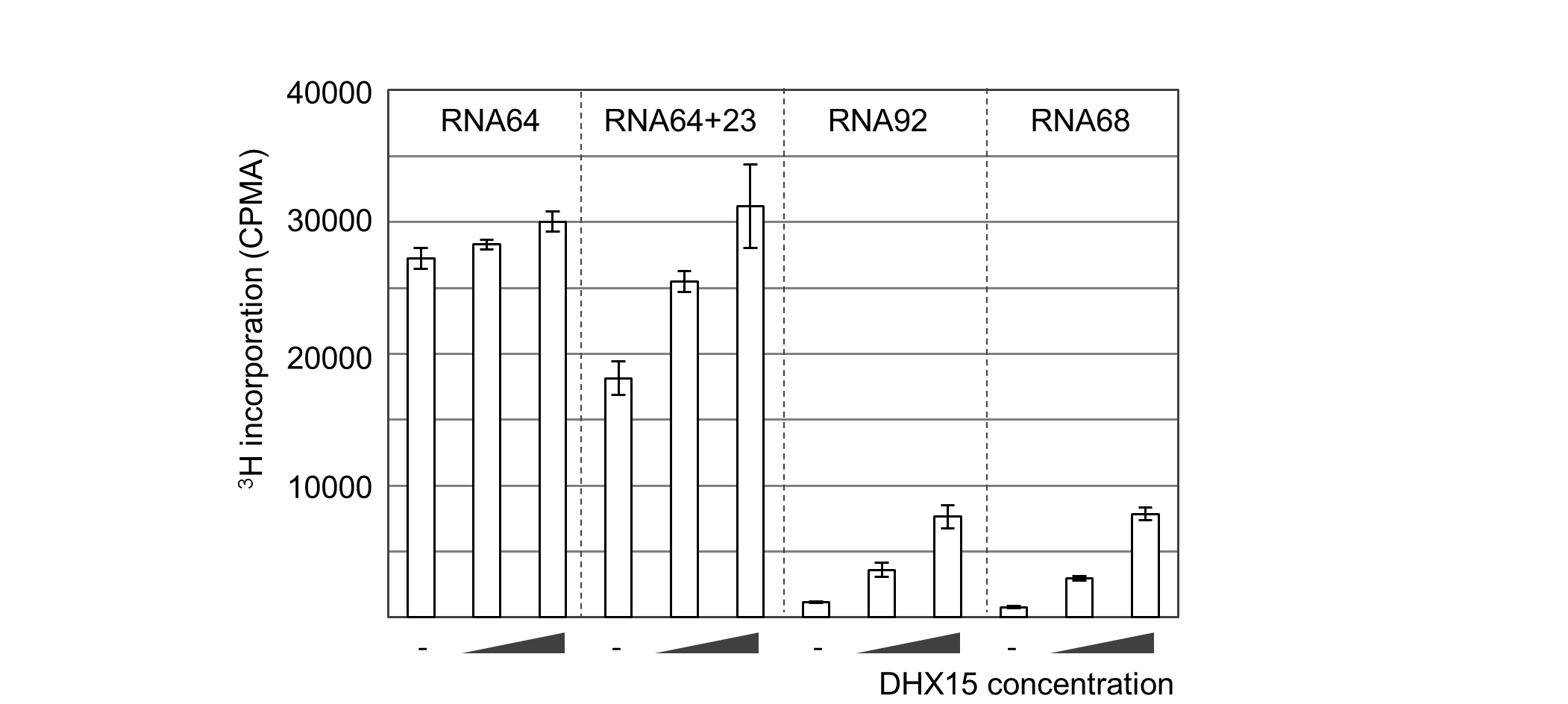


**Supplementary Figure 5.** CMTr1 methyltransferase activity towards different RNA substrates, expressed as a level of tritium-labeled methyl group transfer onto different RNA substrates (shown in Figure 2C), in the absence and presence of DHX15 at 1:1 and 1:5 molar ratios. Results from three independent experiments are shown. Error bars indicate standard deviations.

**
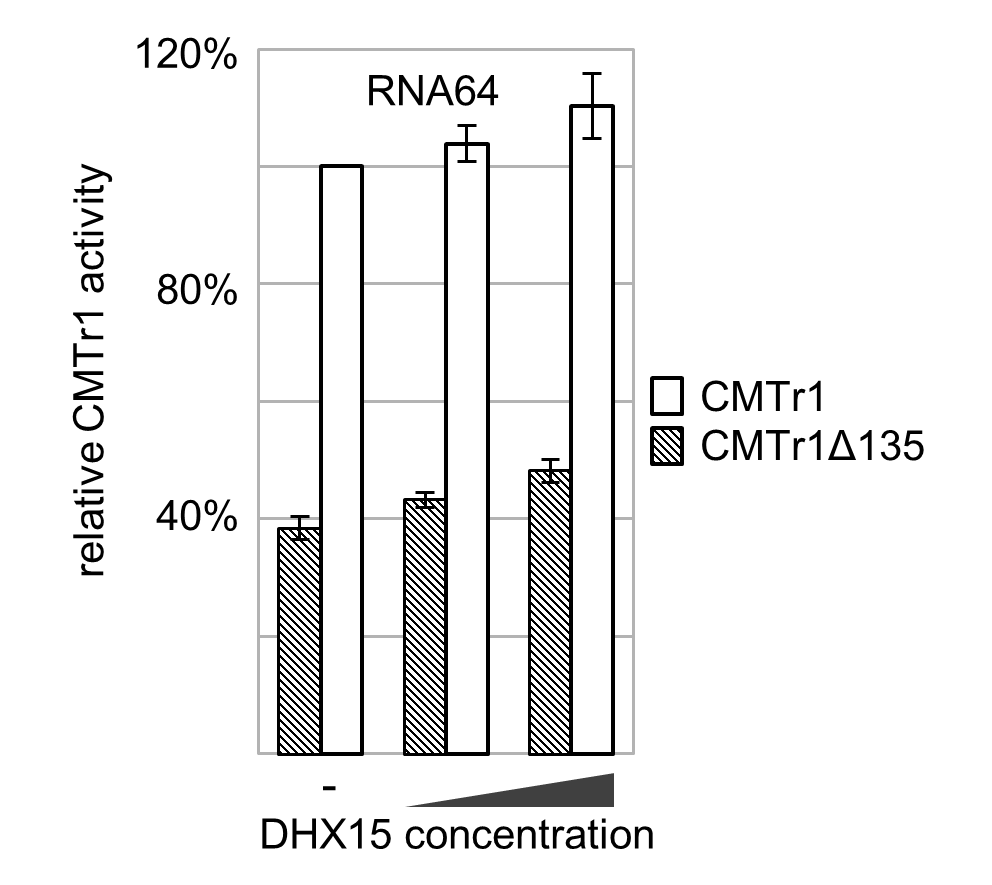
**

**Supplementary Figure 6. Comparison of CMTr1 and CMTr1Δ135 activities on RNA64 substrate in the absence and presence of DHX15**. Results from three independent experiments are shown. Error bars indicate standard deviations. As shown in Figure 3 (main text), for RNA68 (a substrate with a strongly structured 5′ end), the activity of both CMTr1 and CMTr1Δ135 is minimal, and CMTr1 is strongly stimulated by the presence of DHX15. Here, both CMTr1 and CMTr1Δ135 show considerable activity on RNA64 (a substrate with a weakly structured 5′ end), but the stimulatory effect of DHX15 on either protein is minimal.


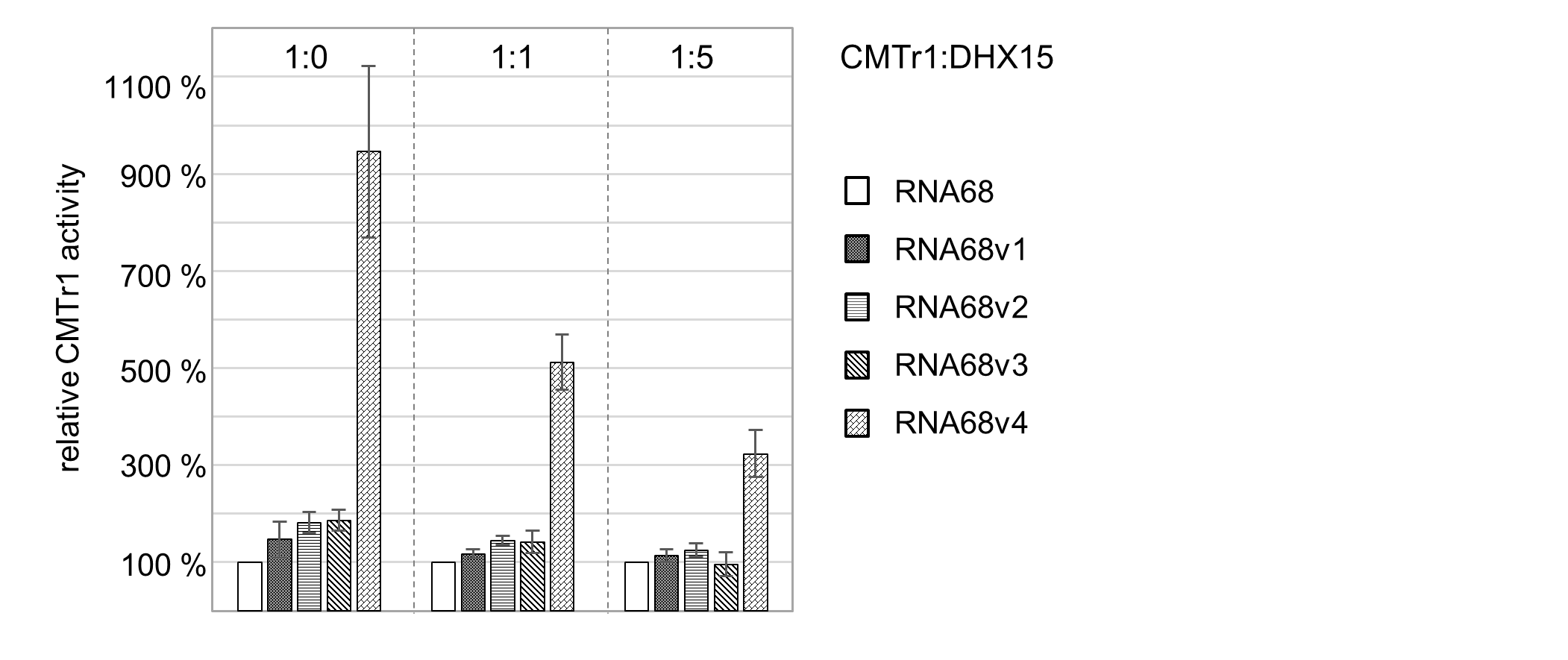


**Supplementary Figure 7. CMTr1 methyltransferase activity towards different RNA substrates normalized to values observed for RNA68 (shown as 100%).** This series of plots illustrates the same data as shown in Figure 4D. This comparison shows the difference in the CMTr1 effectiveness to methylate substrates with the different strength of the secondary structure and emphasizes the scale of DHX15 influence on CMTr1 activity, at 1:1 and 1:5 molar ratios. In the increasing presence of DHX15, the differences in CMTr1 activity on different substrates are reduced.


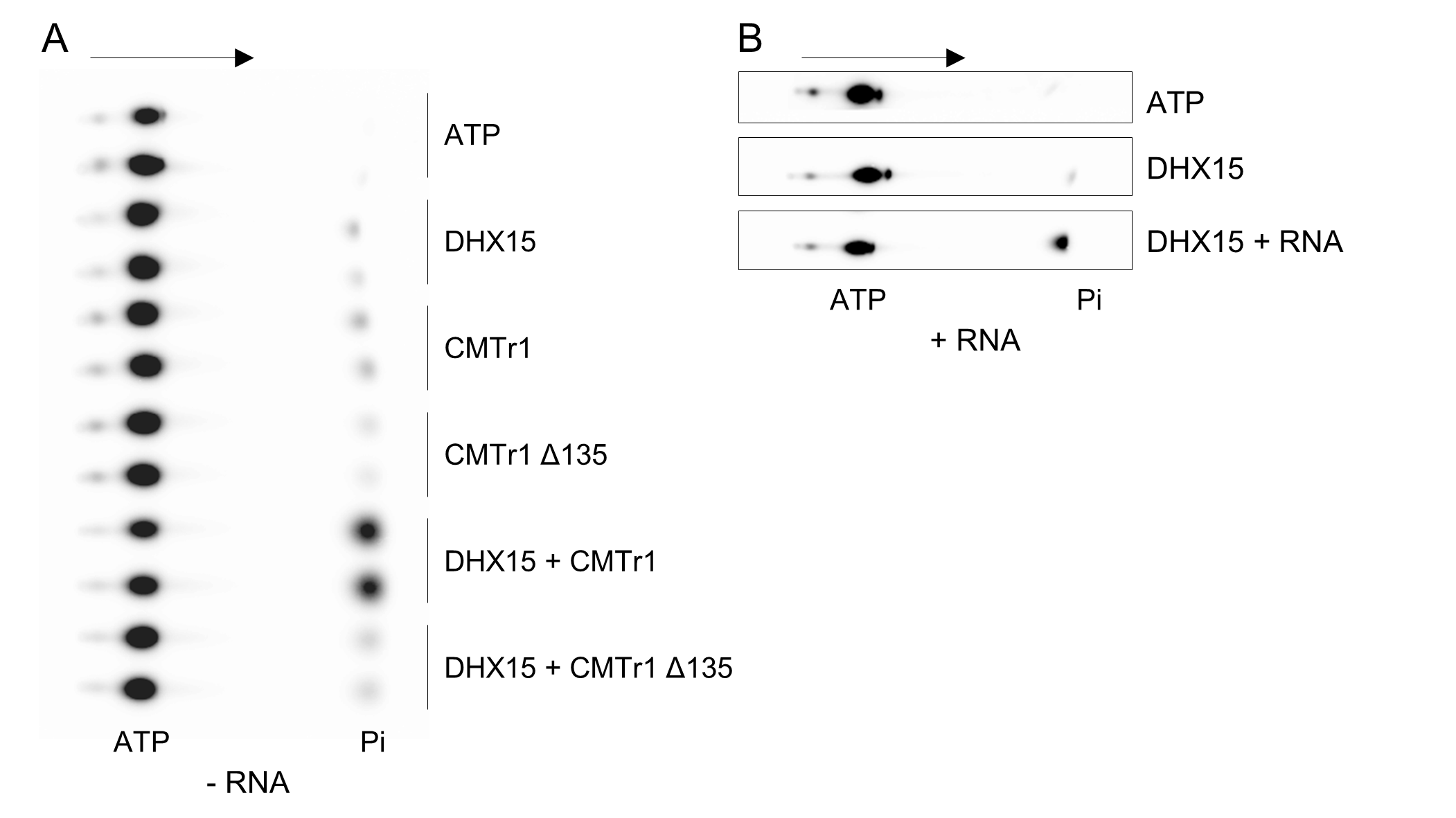


**Supplementary Figure 8. ATPase activity of DHX15 in the presence or absence of CMTr1 methyltransferase at 1:1 ratio.** The ATPase activity of DHX15 at 0.2 μM concentration was measured in a 15 μl reaction volume containing 10 μCi ^32^P-labeled ATP and unlabeled ATP at 50 μM final concentration. The reaction mixtures were incubated at 25°C for 60 min and stopped on ice. 1.5 μl of each sample was analyzed by thin-layer chromatography on polyethyleneimine-cellulose plates (Merck) using 0.1 M potassium phosphate pH 7.4 as migration buffer. The plates were dried, and the radioactivity was quantified on a Typhoon Trio Phosphorimager (GE Healthcare). A) ATP hydrolysis ([α-^32^P]ATP to [α-^32^P]ADP) by DHX15 in the presence of CMTr1 or its variant CMTr1Δ135. To determine the basal ATPase activities, DHX15 and CMTr1 proteins (0.2 μM) were preincubated with RNase A (10 μg/ml) for 10 min at 25°C, to eliminate the possibility that the protein preparations contained contaminating RNA, and then added to the reaction mixtures. Representative autoradiograph of two technical replicates is shown. The arrow indicates resolving direction. B) ATP hydrolysis by DHX15 stimulated by RNA. The RNA-dependent ATPase activity was measured in the presence of 30 μM E.coli tRNA. Representative autoradiograph is shown. The arrow indicates resolving direction.


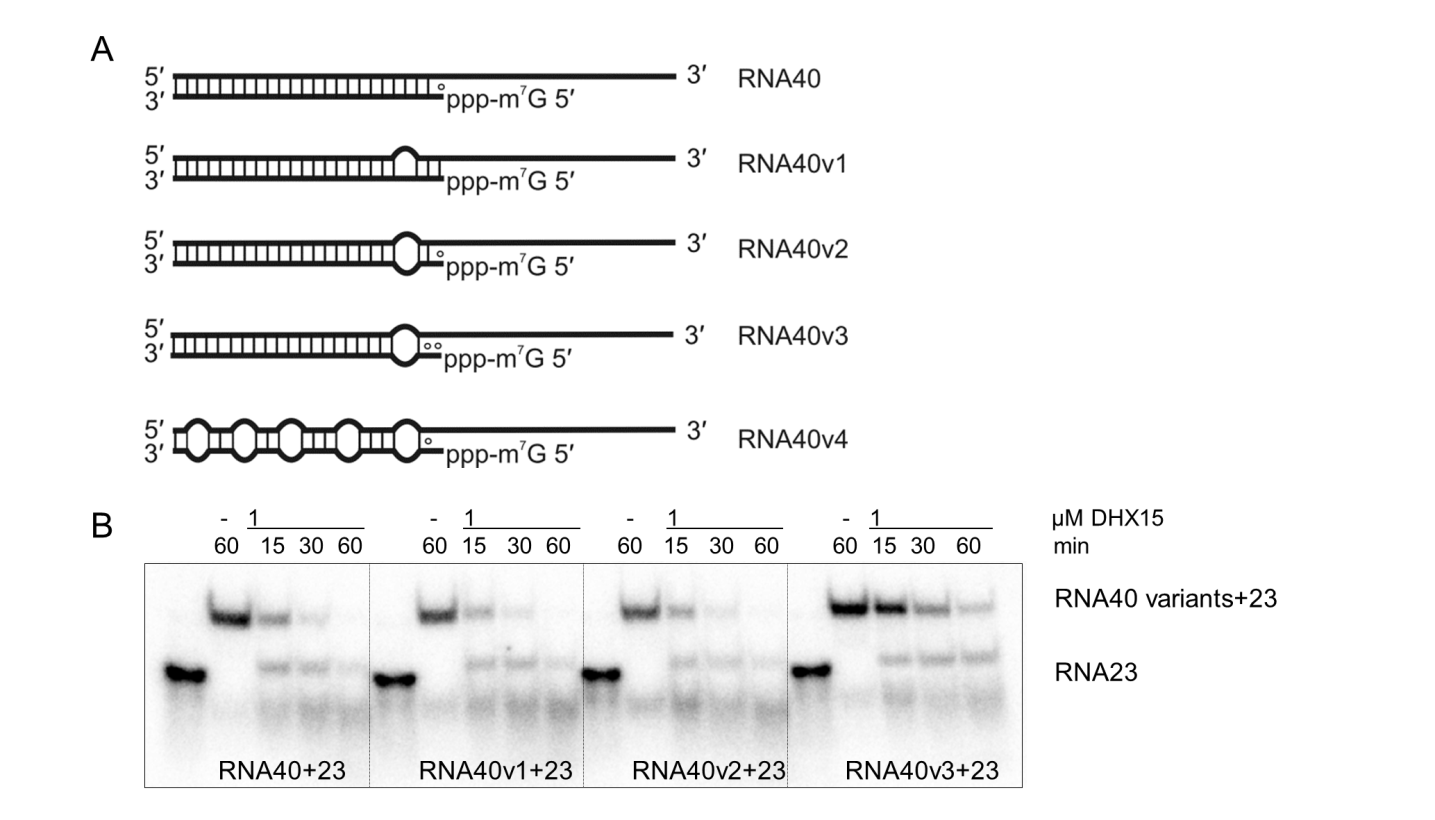


**Supplementary Figure 9. DHX15 activity towards substrates that differ in the strength of secondary structure at their 5**′ **termini.** The helicase activity was monitored by unwinding RNAs with base-paired 5′ ends. RNA40 variants hybridized to ^32^P-labeled RNA23 were used as substrates. Strand displacement reactions containing 10 nM RNA and 1 μM DHX15 were carried out in BDHX buffer supplemented with 0.1 μM ATP in a total volume of 10 μl. At indicated time points, reactions were stopped by addition of equal volume of 6x DNA Loading Dye (ThermoFisher Scientific) supplemented with 20% phenol pH 5.5. RNA was resolved on native 10% polyacrylamide gels, and visualized by autoradiography (Typhoon Trio, GE Healthcare). Gel band intensities were quantified with ImageQuant software (GE Healthcare). A) Schematic representation of RNA substrates; canonical Watson-Crick base pairs a (A-U and G-C) and “○” denotes a wobble pair G-U in the 5′**-**terminal hairpin. Sequences of RNA substrates and their predicted secondary structures are shown in Supplementary Table 2. B) Unwinding RNA/RNA duplexes with the single-stranded 3′ end as a function of time (RNA40+23 - RNA40v3+23). RNA40v4+23 substrate was not included in the assay as the resulting duplex was not stable. Representative autoradiograph of the dried gel is shown.


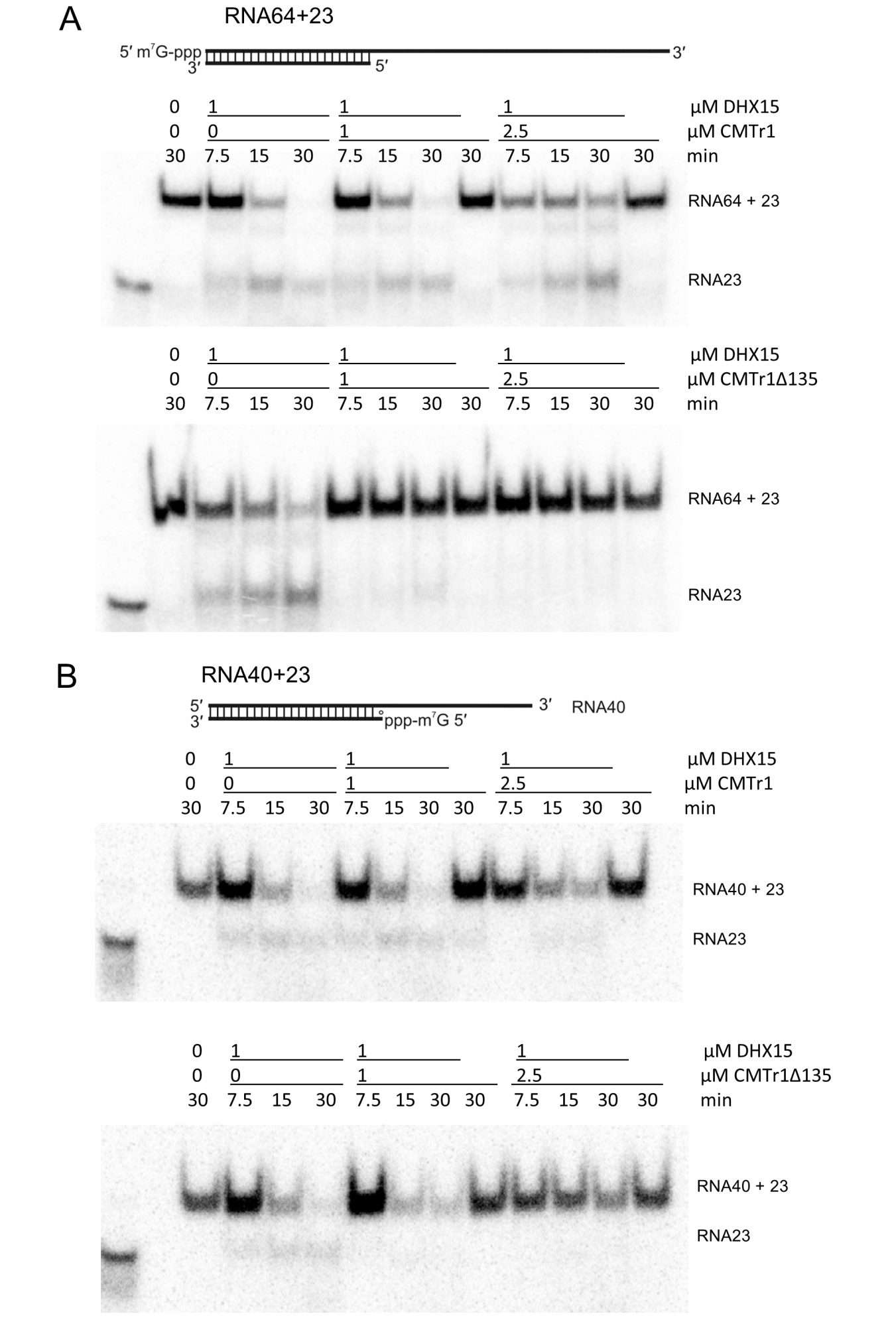


**Supplementary Figure 10. Strand displacement activity of DHX15 towards capped RNA64+23 and RNA40+23 substrates in the presence or absence of CMTr1 methyltransferase at 1:1 and 1:2.5 ratios.** RNA40 and capped-RNA64 hybridized to
^32^P-labeled RNA23 were used as substrates. Strand displacement reactions containing 10 nM RNA, 1 μM DHX15 and optionally 1 to 2.5 μM CMTr1 variants were carried out in BDHX buffer supplemented with 0.1 μM ATP in a total volume of 10 μl. At indicated time points, reactions were stopped by addition of equal volume of 6x DNA Loading Dye (ThermoFisher Scientific) supplemented with 20% phenol pH 5.5. RNA was resolved on native 10% polyacrylamide gels and visualized by autoradiography (Typhoon Trio, GE Healthcare). Gel band intensities were quantified with ImageQuant software (GE Healthcare). The unwinding of capped (A) and not capped (B) RNA/RNA duplexes with the single-stranded 3′ end as a function of time. Representative autoradiographs of the dried gels are shown. Schematic representation of RNA substrates are shown above autoradiographs. Canonical Watson-Crick base pairs (A-U and G-C) are indicated and “○” denotes a wobble pair G-U in the 5′**-**terminal hairpin. Sequences of RNA substrates and their predicted secondary structures are shown in Supplementary Table 2 and Supplementary Figure 2.

**
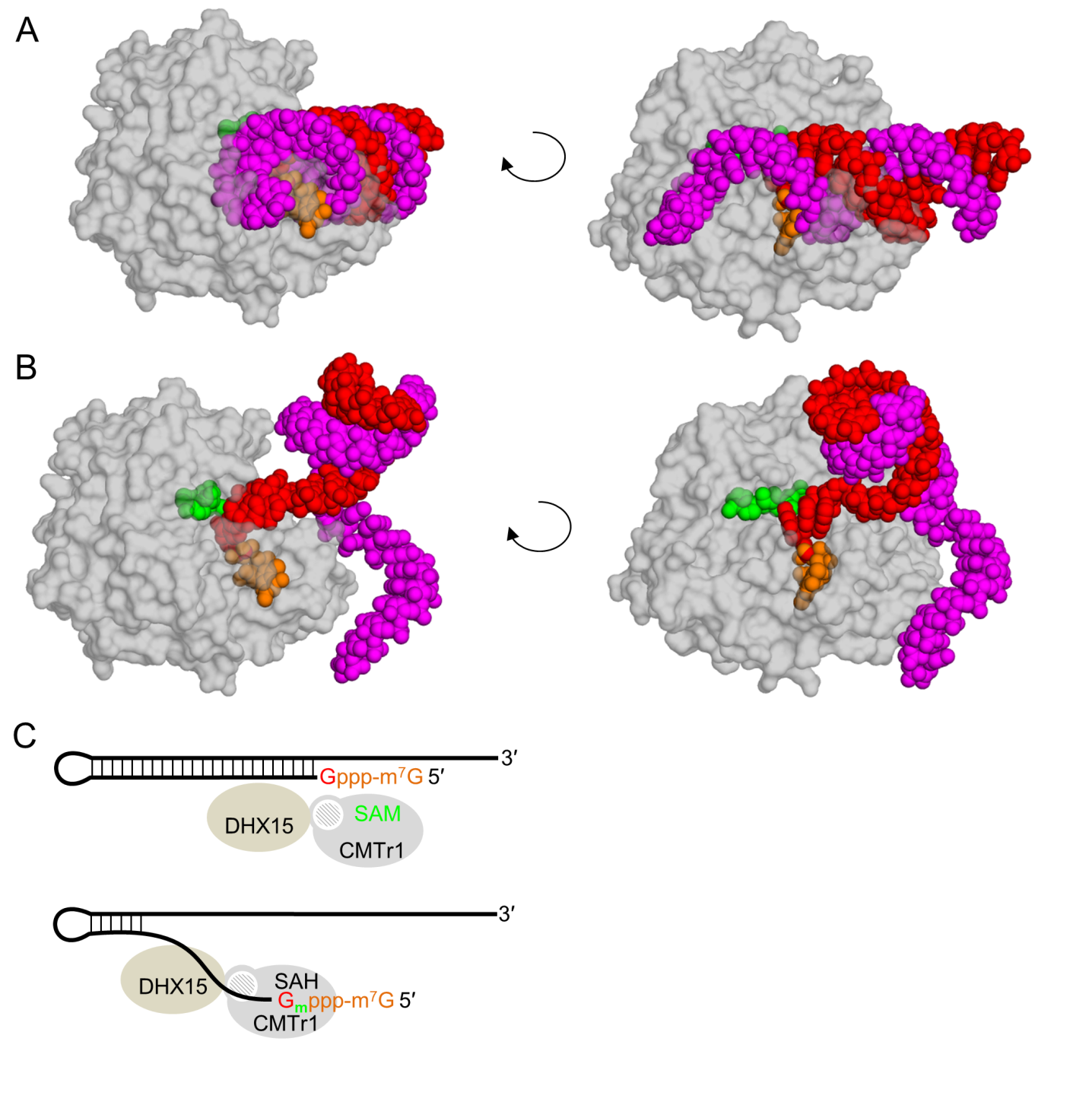
**

**Supplementary Figure 11. Proposed model of CMTr1-DHX15 cooperation for efficient methylation of transcripts with structured 5′ ends.** A) and B) 3D structural model of CMTr1 catalytic domain (PDB ID: 4N48; light grey, semi-transparent) in complex with the substrate RNA (different strands in red and magenta, respectively, m^7^G cap in orange) and SAM (green). CMTr1 in complex with SAM, m^7^G and four 5′**-**proximal residues correspond to the crystal structure [9] and are identical between A) and B), while the remaining parts of RNA are speculative and chance from A) to B). A) Implausible interaction of CMTr1 with fully dsRNA fully-base-paired at the 5′ end, which leads to steric clashes (red and magenta RNA overlaps with the semi-transparent grey protein). Clashes are extensive for the base-paired 5′**-**proximal double-stranded segment (RNA in red and magenta), and additional steric conflicts appear for the single-stranded 3′-proximal region (RNA in magenta). B) Plausible interaction of CMTr1 with partially unpaired RNA, which minimizes steric clashes. As evident from the image, further unwinding/unpairing of dsRNA would move the substrate away from CMTr1, minimizing the likelihood of steric conflicts. C) Schematic diagram illustrating CMTr1 cooperation with DHX15, illustrating structural changes in dsRNA that enable CMTr1 access to 5′ cap.

**SUPPLEMENTARY REFERENCES**

1. Haline-Vaz T, Silva TC, Zanchin NI. (2008) The human interferon-regulated ISG95 protein interacts with RNA polymerase II and shows methyltransferase activity. Biochem Biophys Res Commun. 2008 Aug 8;372(4):719-24.
2. Mosallanejad K, Sekine Y, Ishikura-Kinoshita S, Kumagai K, Nagano T, Matsuzawa A, Takeda K, Naguro I, Ichijo H. (2014) The DEAH-box RNA helicase DHX15 activates NF-κB and MAPK signaling downstream of MAVS during antiviral responses. Sci Signal. 2014 Apr 29;7(323):ra40.
3. Li R, Zhang H, Yu W, Chen Y, Gui B, Liang J, Wang Y, Sun L, Yang X, Zhang Y, Shi L, Li Y, Shang Y. (2009) ZIP: a novel transcription repressor, represses EGFR oncogene and suppresses breast carcinogenesis. EMBO J. 2009 Sep 16;28(18):2763-76.
4. Niu Z, Jin W, Zhang L, Li X. Tumor suppressor RBM5 directly interacts with the DExD/H-box protein DHX15 and stimulates its helicase activity. FEBS Lett. 2012 Apr 5;586(7):977-83.
5. Wilkinson KA, Merino EJ, Weeks KM. Selective 2’‐hydroxyl acylation analyzed by primer extension (SHAPE): quantitative RNA structure analysis at single nucleotide resolution. Nat Protoc 2006, 1:1610–1616.
6. Duncan CD1, Weeks KM. SHAPE analysis of long-range interactions reveals extensive and thermodynamically preferred misfolding in a fragile group I intron RNA. Biochemistry. 2008 Aug 19;47(33):8504-13.
7. Karabiber F, McGinnis JL, Favorov OV, Weeks KM. QuShape: rapid, accurate, and best-practices quantification of nucleic acid probing information, resolved by capillary electrophoresis. RNA. 2013 Jan;19(1):63-73.
8. McGinnis JL, Duncan CD, Weeks KM. High-throughput SHAPE and hydroxyl radical analysis of RNA structure and ribonucleoprotein assembly. Methods Enzymol. 2009;468:67-89.
9. Smietanski M, Werner M, Purta E, Kaminska KH, Stepinski J, Darzynkiewicz E, Nowotny M, Bujnicki JM. Structural analysis of human 2'-O-ribose methyltransferases involved in mRNA cap structure formation. Nat Commun. 2014;5:3004.
